# Systematic Validation and Atomic Force Microscopy of Non-Covalent Short Oligonucleotide Barcode Microarrays

**DOI:** 10.1371/journal.pone.0001546

**Published:** 2008-02-06

**Authors:** Michael A. Cook, Chi-Kin Chan, Paul Jorgensen, Troy Ketela, Daniel So, Mike Tyers, Chi-Yip Ho

**Affiliations:** 1 Centre for Systems Biology, Samuel Lunenfeld Research Institute, Mount Sinai Hospital, Toronto, Ontario, Canada; 2 Department of Molecular Genetics, University of Toronto, Toronto, Ontario, Canada; 3 Microarray Laboratory, Samuel Lunenfeld Research Institute, Mount Sinai Hospital, Toronto, Ontario, Canada; 4 Terrence Donnelly Center for Cellular and Biomolecular Research, University of Toronto, 1Toronto, Ontario, Canada; 5 Scenterra Inc., Bowie, Maryland, United States of America; Cairo University, Egypt

## Abstract

**Background:**

Molecular barcode arrays provide a powerful means to analyze cellular phenotypes in parallel through detection of short (20–60 base) unique sequence tags, or “barcodes”, associated with each strain or clone in a collection. However, costs of current methods for microarray construction, whether by *in situ* oligonucleotide synthesis or *ex situ* coupling of modified oligonucleotides to the slide surface are often prohibitive to large-scale analyses.

**Methodology/Principal Findings:**

Here we demonstrate that unmodified 20mer oligonucleotide probes printed on conventional surfaces show comparable hybridization signals to covalently linked 5′-amino-modified probes. As a test case, we undertook systematic cell size analysis of the budding yeast *Saccharomyces cerevisiae* genome-wide deletion collection by size separation of the deletion pool followed by determination of strain abundance in size fractions by barcode arrays. We demonstrate that the properties of a 13K unique feature spotted 20 mer oligonucleotide barcode microarray compare favorably with an analogous covalently-linked oligonucleotide array. Further, cell size profiles obtained with the size selection/barcode array approach recapitulate previous cell size measurements of individual deletion strains. Finally, through atomic force microscopy (AFM), we characterize the mechanism of hybridization to unmodified barcode probes on the slide surface.

**Conclusions/Significance:**

These studies push the lower limit of probe size in genome-scale unmodified oligonucleotide microarray construction and demonstrate a versatile, cost-effective and reliable method for molecular barcode analysis.

## Introduction

DNA microarray technology has become a standard component in the toolbox of molecular biology. Microarrays have been applied to genome-wide analysis of gene expression, location of transcription factor binding sites (chromatin immunoprecipitation on microarray chip, ChIP-chip), DNA replication fork progression, sister chromatid cohesion, and nucleosome phasing [1]–[5]. More recently, molecular barcode arrays have been used for phenotypic profiling, drug sensitivity and systematic synthetic lethal analysis [6]–[12]. These microarray-based methods facilitate the prediction and definition of gene function, and have broad application in drug discovery and development.

Microarray technology relies on the hybridization of a labeled target sequence to a complementary cDNA or oligonucleotide probe immobilized on a glass surface. The method of deposition and immobilization varies depending upon the average length of the probe. For cDNA and long oligonucleotide sequences, probes produced *ex situ* are typically spotted onto a positively charged surface, such as poly-lysine or amino-silane, and are immobilized through UV cross-linking [13]–[15]. Covalent bond formation is thought to occur primarily through thymine bases in the DNA probes [16], [17]. However, in the case of shorter oligonucleotides (15–60 mer), which possess a smaller complementary sequence over which to bind their cognate targets, probes are commonly synthesized with a 5′-chemically reactive linker [18]. The linker serves to introduce physical distance between the probe and the glass surface, thereby reducing steric hindrance during hybridization, and to allow covalent coupling of the probe to the derivatized surface of a slide via its reactive thiol or amino group, rather than an internal nucleotide base. In a more sophisticated approach, probes can be synthesized *in situ* on the array surface using ink-jet or light-directed oligonucleotide synthesizers, thereby bypassing the need for a secondary linkage reaction [19], [20]. The complications of *in situ* synthesis or *ex situ* derivatization of oligonucleotides add considerable expense to the fabrication process, particularly when hundreds of high-density microarrays are required.

To ameliorate the cost of array fabrication, small unmodified oligonucleotides have been successfully spotted on conventional surfaces [13], [21], [22] or on surfaces modified for better adsorption of molecules [23] on both trial and genome-wide scales [23], [24]. However, under commonly used hybridization conditions, as probe size is reduced below ∼40 bases, hybridization efficiencies have been shown to drop precipitously [13], [21]. Recent small-scale application of reactive poly-carbodiimide surface substrates has enabled use of the smallest yet unmodified oligonucleotide probes (10–12 mer) [25], [26]; however, the performance of this system on a genome-wide scale, with the corresponding large dynamic range of target abundances, hybridization efficiencies, and probe sequence compositions has yet to be ascertained.

Rather than applying newly introduced microarray surface substrates, we optimized a method to spot and hybridize unmodified short 20 mer oligonucleotide probes on conventional amino-silane based microarray surfaces. We applied the method to construct a 12,683 unique feature array that is complementary to the barcode tags of the budding yeast deletion strain collection. This collection, constructed by an international consortium, is composed of ∼6000 individual yeast strains that bear precise null deletions of each known or predicted open reading frame (ORFs) [27]. Each deletion construct in the collection is flanked by two 56 bp cassettes, which are comprised of universal primer sequences flanking a unique 20 mer DNA sequence identifier referred to as a barcode. The barcode tags enable genome-wide profiles of pooled populations to be assessed in a single experiment. In a typical experiment, DNA is extracted from the pooled population before and after selection, barcode sequences are amplified and differentially labeled, and the degree of enrichment or depletion of each strain in the selected population is determined by barcode microarray analysis.

Here, we demonstrate that short unmodified oligonucleotide probes spotted on Corning GAPS™II slides yield comparable signal intensities and signal-to-noise ratios (SNRs) to 5′-amino-modified covalently linked oligonucleotides. In a proof-of-concept application, we use a 13K feature barcode microarray to analyze the size distribution of the entire yeast deletion collection in a single experiment. By direct comparison of these experiments with previously obtained genome-scale cell size data [28], we provide rigorous biological validation of our unmodified oligonucleotide arrays and of the barcode approach to cell size determination. Further, as part of our analyses, we outline methodology to minimize false discovery and to define significant enrichment. We demonstrate that data obtained from short unmodified oligonucleotide arrays, while not equally precise as that obtained using inkjet-synthesized Agilent barcode arrays, are nonetheless specific, and as biologically accurate and comprehensive. Lastly, we use atomic force microscopy (AFM) to examine the arrangement of spotted 20 mer oligonucleotides, both before and after binding of their cognate target sequences. Through this we provide evidence as to the mechanism of target hybridization to unmodified oligonucleotide probes at a molecular level. The properties of short unmodified oligonucleotide arrays provide a considerable cost-saving alternative in barcode or short oligonucleotide DNA microarray fabrication, particularly amenable to large-scale in-house synthesis efforts (in our case, forgoing oligonucleotide modification confers an approximately 75% savings in synthesis costs; Supplementary Table S1). The savings imparted can, in turn, be passed on to other laboratories, providing greater access to barcode microarray technology.

## Results

### Performance of amino-modified short oligonucleotide 20 mer probes

**Figure 1:**
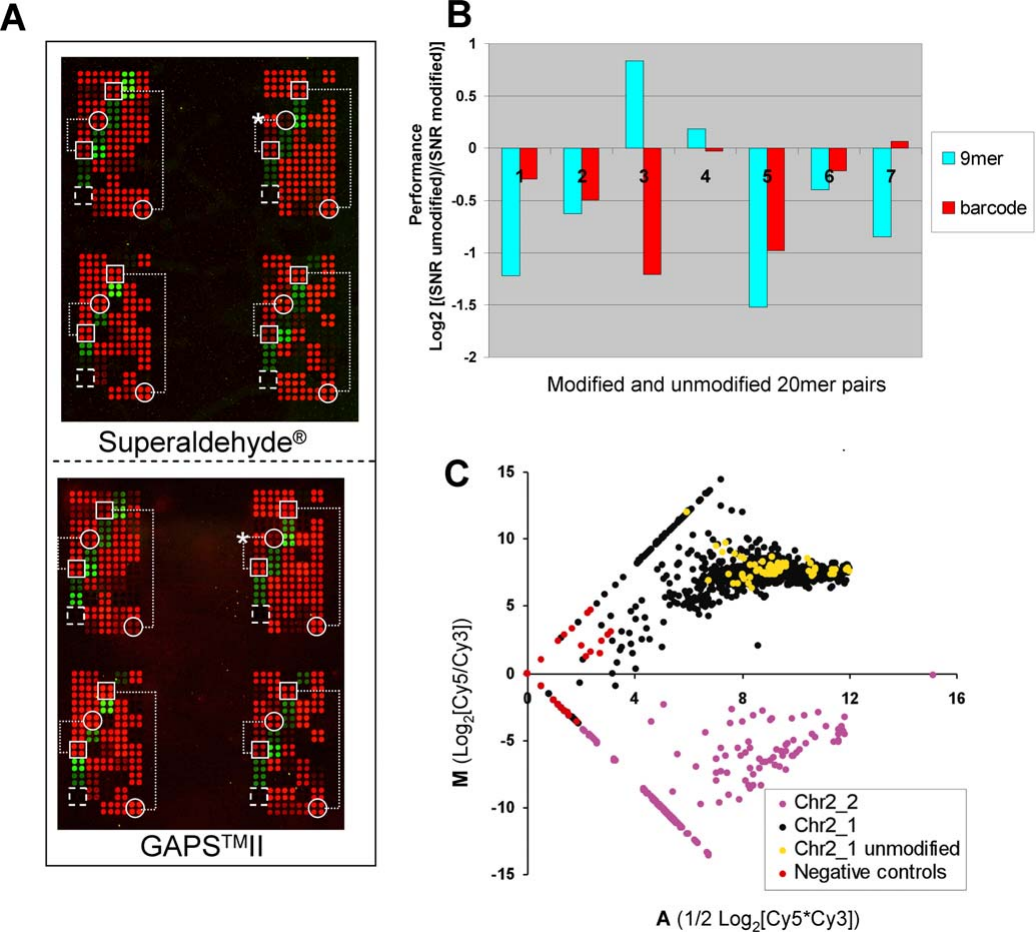
Sensitivity and specificity of spotted 20mer barcode oligonucleotides. A. Fluorescent images of hybridized pilot barcode arrays fabricated on Superaldehyde® (top; substrate permissive to covalent linkage of probes) and GAPS™II (bottom; substrate non-permissive to covalent linkage of probes) slides. 184 barcode oligonucleotides were printed as two sets of quadruplet features in each of two identical arrays (or “super-grids”; only the top super-grid is shown for each substrate). Pairs of amino-modified (white circles) and unmodified control probes (white squares) are shown linked by dotted lines. A defective modified barcode, excluded from further analysis, is noted (white asters). Negative controls are shown (white dashed box). All other unmarked features hybridized with barcodes from sub-populations of non-essential deletion strains of chromosome 2, labeled with either Cy5 (Chr2_1) or Cy3 (Chr2_2). B. Effect of 5′-amino-modification on oligonucleotide performance. Performance of each of seven different barcode oligonucleotide pairs (sequences are provided in a Supplemental GeneList file; List Data S1; the probe pairs correspond to YBL090W-DN, YBL091C-DN, YBL091C-UP, YBL093C-DN, YBL093C-UP, YBL094C-DN, YBL094C-UP, from left to right) is measured by log2 ratio of SNR(unmodified)/SNR(modified) on Superaldehyde® substrate, corrected by subtracting the log2 ratio of that on GAPS™II substrate; where SNR denotes signal to noise ratio of the probe [(median intensity Cy5 [or Cy3] - median background Cy5 [or Cy3])/standard deviation background Cy5 (or Cy3)]. Performance was determined based on hybridization to the cognate barcode sequences (barcode, from Figure 1A) or to a Cy3-labeled randomized 9mer probe. C. Specificity and signal intensity of hybridized barcodes in the absence of covalent linkage to substrate. The log2 ratio of the background subtracted Cy5/Cy3 channels (M) is plotted versus the average log2 value of the signal intensity in each channel (A) for 8 replicates of each barcode. Barcodes from sub-populations of non-essential deletion strains from chromosome 2 are labeled with Cy5 (Chr2_1; black [amino-modified] and gold [unmodified]) and Cy3 (Chr2_2; magenta). Negative control sequences are shown (red). Spots with background-subtracted intensities below zero for one channel fall along straight lines. Negative values were assigned an arbitrary floor value of 1 (log2 = 0) in calculations of A and M. (The fluorescent image of 4 replicates for each probe is shown in the bottom panel of Figure 1A.)

Prior to constructing whole genome yeast barcode arrays, we compared performance of arrays prepared by different methods. We designed a pilot array containing 5′-amino modified 20 mer probes for both UP and DOWN (hereafter “DN”) tags of 92 non-essential open reading frames (ORFs) from chromosome 2 (total of 184 unique probe sequences). To assess the effect of the 5′ modification reaction, we compared the hybridization properties of eight control probes lacking the 5′-modification versus their modified counterparts on two substrates that did or did not supply reactive groups for covalent bonding, namely SuperAldehyde® (Telechem) and GAPS™II (Corning), respectively. We prepared barcode PCR products from two pools of yeast deletion strains: barcode tags from the first pool (EUROSCARF Chr 2_1, comprised of 75 strains and their 150 universal barcodes tags), were amplified and labeled with Cy5 fluorescent primers, while barcode tags from the second pool (EUROSCARF Chr2_2, comprised of 76 deletion strains, of which only 15 ORFs and a total of 30 tags were represented on the array), were amplified and labeled with Cy3. The remaining four barcode probes of the pilot array served as negative controls that had no labeled targets. On both Superaldehyde® and GAPS™II substrates, the barcode features displayed either red (Cy5) or green (Cy3) but not yellow (mixed) colors, thus representing their cognate fluorescent signals without detectable cross-hybridization (Figure 1A). Overall signal intensities were comparable regardless of the substrate used. Negative controls showed only background intensities (dashed boxes). The differences in signal intensity between 5′-amino-modified (circled features) and unmodified control spots (boxed features) on the Superaldehyde® surface were indistinguishable.

To determine the effect of covalent linkage on hybridization efficiency, we compared the performance of unmodified versus modified 20 mer probes, as the ratio of the two fractions, namely [(signal of unmodified probe)/(signal of modified probe)] on Superaldehyde® over that of GAPS™II substrate. The latter fraction would reflect (and adjust for) differences due to idiosyncrasies in the printing and synthesis, including the quality and quantity of oligonucleotide synthesized and any positional effects during printing, as opposed to direct effects of covalent linkage. One probe pair, marked with white asters (Figure 1A), showed substantially lower hybridization signal for the modified probe even on the GAPS™II substrate, suggesting a problem during synthesis. Consistently, mass spectrometric (MS) analysis showed an aberrant mass in the case of the modified probe, but not of its unmodified counterpart (not shown). As such, we excluded this probe pair from our analysis. All other seven pairs of oligonucleotides had expected masses. To compare hybridization signals of different probes on different substrates, we used SNR instead of raw fluorescent intensities in calculations of their performances. To eliminate any bias caused by target sequence length, we determined the performance of probes in hybridization to both labeled 56 mer barcode amplicons and Cy3-labeled random 9 mers. The results of both of these analyses revealed that the unmodified probes yielded a signal on average of 0.7 fold of the corresponding modified probes (Figure 1B).

Given the unexpectedly modest advantage conferred by 5′-modification and covalent linkage, we further characterized the performance of modified probes on GAPS™II slides. As expected, SNRs for modified and unmodified probes were approximately equal for analogous probe sequences (not shown). In a plot of log2 ratio (M) of the Cy5 and Cy3 signals versus the average log2 intensity (A) of the two channels (Figure 1C), we observed no significant cross-hybridization between barcode signals from the two sub-populations of deletion strains. Both sub-populations showed specific (|log2 ratio| >2) signals for their respective labels, with the exception of 1 (of 8) replicate spots for each of two barcode probes; unmodified probes (gold circles) showed comparable specificity to their amino-modified counterparts. 83% of barcode tags displayed 3-fold higher signal than background; moreover, only one gene (∼1% of probes) failed for both barcodes. This detection rate is unexpectedly high, given that re-sequencing has revealed that ∼31% of all barcode tags are different from those originally designed, and approximately 25% have anomalous hybridization properties for at least one barcode [29]. When we examined the barcode probes listed as anomalous, we found that the majority actually had signal intensities comparable to correct barcodes, suggesting that performance may be improved by non-covalent array construction (Figure S1). In summary, from this pilot comparison, we concluded that non-covalently linked 20mer barcode probes spotted on a GAPS™II substrate perform at least as well as covalently coupled probes.

### A 13K genome-wide yeast barcode array

**Figure 2:**
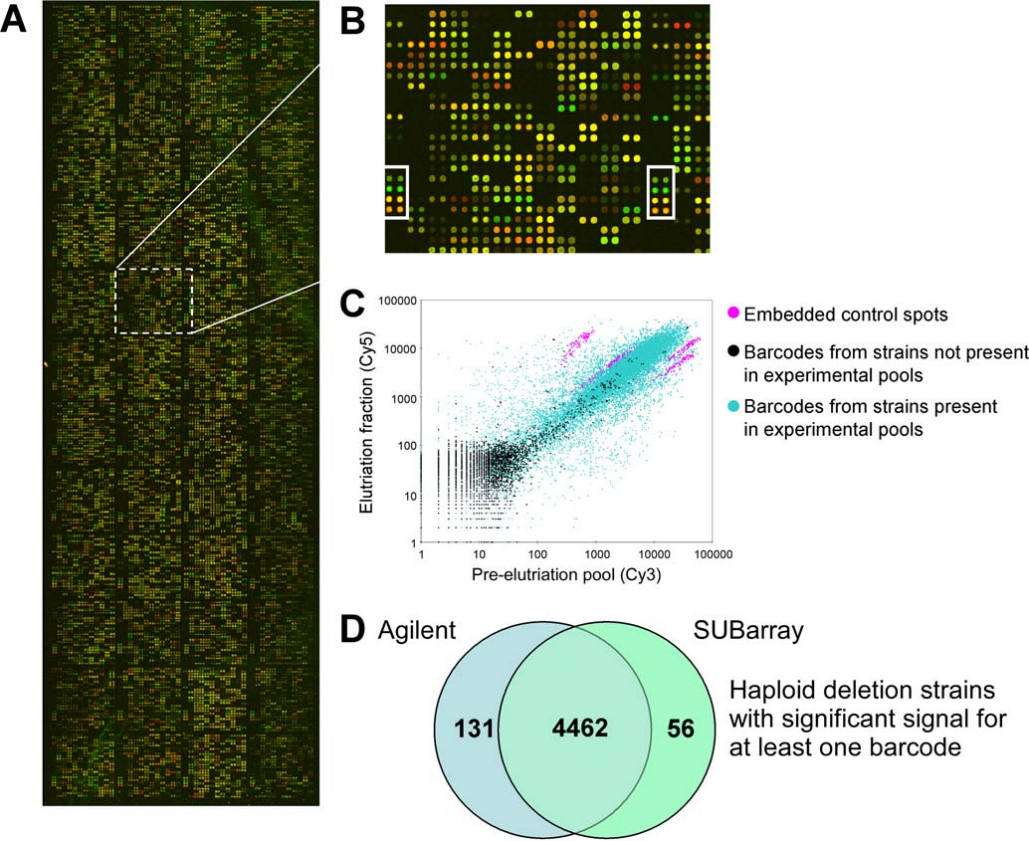
Features of the 13K unique feature SUBarray. A. A representative microarray from an experiment with the haploid yeast deletion set. Arrays are constructed of 48 blocks. B. Enlargement of a region overlapping two of the blocks. In white boxes are the four oligonucleotide controls that are present within every block. C. Logarithmic scale scatter plot of background subtracted intensity for Cy5 versus Cy3-dye for a representative barcode elutriation cell size experiment. Barcodes of strains not present within the experimental pool (black) and the four sets of positive controls (magenta) are overlayed on the barcodes of strains represented in the pool (blue). D. Overlap of strains represented by at least one significant barcode signal between Agilent covalently linked 20mer arrays and SUBarrays. Significant barcode signals are as defined in the text.

Given the above results with a pilot barcode microarray, we constructed a short unmodified barcode probe array with 13K unique features (which hereafter is referred to as the “SUBarray”). The complete set of barcodes in the yeast deletion set was synthesized commercially at low cost (Illumina) and spotted in duplicate on GAPS™II slides (Figure 2A). In addition to the barcodes initially described in the yeast deletion project [27], we included 896 updated probe sequences to correct errors in the deletion cassettes themselves [29]. We also included 4 control pairs (2 UP tags and 2 DN tags) within each of the 48 printed blocks of probes (Figure 2B, 2C). These control probes were chosen for their consistent detection across a broad range of experimental conditions (not shown) and serve as a measure of consistent hybridization efficiency across the surface of the array.

Not surprisingly, given the novel construction of our array, we found that optimal performance required very different hybridization conditions than previously published for other covalent barcode microarray constructions [30]. In particular, lowering the temperature to 25°C, changing the hybridization buffer (DIG Easy Hybe, Roche), and decreasing the hybridization volume by applying sample under a raised coverslip (LifterSlip, Erie Scientific) drastically increased signal intensities, lowered background, and reduced false signals (not shown).

To initially characterize the SUBarray, we performed an analysis of the complete haploid *MAT* ***a*** yeast deletion pool. As approximately 20% of yeast genes are essential and absent from the haploid collection, the cohort of barcodes from essential genes allowed estimation of both false positive rate (all barcodes absent from pools) and true positive rate (all barcodes present in pools). Greater than 95% of barcode signals for essential gene deletions, and other negative controls, clustered in the low intensity end of a Cy5- versus Cy3-dye intensity scatter plot (Figure 2C, signal intensity for both channels <200) of a representative barcode elutriation cell size experiment (detailed in the next subsection); elutriated sample was labeled with Cy5 versus control or pre-elutriated sample labeled with Cy3. We then used essential gene barcodes to develop a two-step filter for signal significance. In the first step, we applied an SNR threshold on an individual probe basis, choosing the threshold such that false positives were minimized and true positives were maximized. This point is represented by the 45° tangent to a receiver operating characteristic (ROC) curve, at which the rates of loss of false positive and true positive spots are equal (Figure S2A). Comparable thresholds were obtained when different measures of microarray quality were used (Figure S2B). In a second step, we assessed the number of times a given barcode was significant across multiple replicates and multiple arrays, again using the criteria of equal rates of false positive and true positive loss (Figure S2C). We reasoned that true positive barcode signals at near-noise levels should occur above background more frequently than random false positive signals. Using this filter, we recovered only 4.0% (89) of all possible false positive barcodes but captured 83.3% (7835) of all possible true positive barcodes (Table 1). Filtering according to the total number of barcode replicate spots detected across both UP and DN tags further improved resolution between false positive and true positive data (Figure S2D). By this criterion, we identified 5.0% (57) of all possible false positive deletion strains and 94.4% (4518) of all possible true positive deletion strains (Table 1). A detailed description of these methods is provided in the Supplementary material (Supplemental Text S1, Supplemental Charts S1). Finally, gene coverage on the SUBarray was comparable to and overlapped significantly with an Agilent array of covalently coupled barcode DNA oligonucleotides that represented the entire haploid deletion collection (Table 1 and Figure 2D). A detailed description of this covalent barcode array is provided in the Materials and Methods section. Across both technical and biological replicates, the SUBarray experiments yielded a Pearson's correlation coefficient of 0.7 (Table 1). The Agilent arrays we used displayed better correlation between replicate experiments for most barcodes than displayed by the SUBarrays (Supplemental text S1; Figure S5); however, due to the presence of a number of anti-correlated barcodes in these Agilent experiments, the correlation coefficient between dye-swap replicates had a similar value of 0.7 (Table 1). The average log2 transformed Cy5/Cy3 ratios for these anti-correlated barcodes across replicate arrays were approximately zero. Comparison of the average Z scores, meaning the standardized results of the log2 transformed Cy5/Cy3 ratios, across all replicate experiments (similar to the aforementioned cell size elutriation) between the SUBarray (n = 6) and the Agilent array (n = 4), yielded a correlation coefficient of 0.92. Characteristic scatter plots for SUBarrays and Agilent arrays, as well as a comparison between the two platforms, are provided in the Supplementary materials (Figures S4, S5, S6).

**Table 1:** Coverage, specificity, and reproducibility of unmodified 20mer arrays.

*Coverage (True positive/False negative rate* 1 *)*	*SUBarray*	*Agilent*
Barcodes	7835 (83.3%/16.7%)	8031 (85.9%/14.1%)
Gene deletion strains	4518 (94.4%/5.6%)	4593 (96.0%/4.0%)
***Specificity (False positive/True negative rate*** 1 ***)***	*SUBarray*	*Agilent*
Barcodes	89 (4.0%/96.0%)	not applicable^2^
Gene deletion strains	57 (5.0%/95.0%)	not applicable^2^
***Reproducibility*** * Correlation coefficient* 3	*SUBarray*	*Agilent*
Technical replicates	0.75	0.95^5^
Dye swap replicates	0.72	0.71^6^
Intraexperimental biological replicates	0.73	0.82
Comparison with Agilent arrays^4^	0.92	

1 True positive/false negative rates and false positive/true negative rates are expressed in brackets.

2 Since these Agilent arrays lacked barcodes for essential genes, estimates of false positives were not possible.

3 Correlation coefficients represent an average of all possible pair-wise comparisons within the indicated replicate type. Scatter plots are provided in the Supplementary material (Figures S4, S5, S6).

4 Comparison using average of all SUBarrays (n = 6) or Agilent arrays (n = 4).

5 These amplifications used highly similar biological replicates with the same dye-labeling scheme; however, by definition these are not technical replicates.

6 The lower correlation coefficient reflects the presence of numerous dye-swap artifacts.

### Application of short oligonucleotide barcode arrays to cell size control

**Figure 3:**
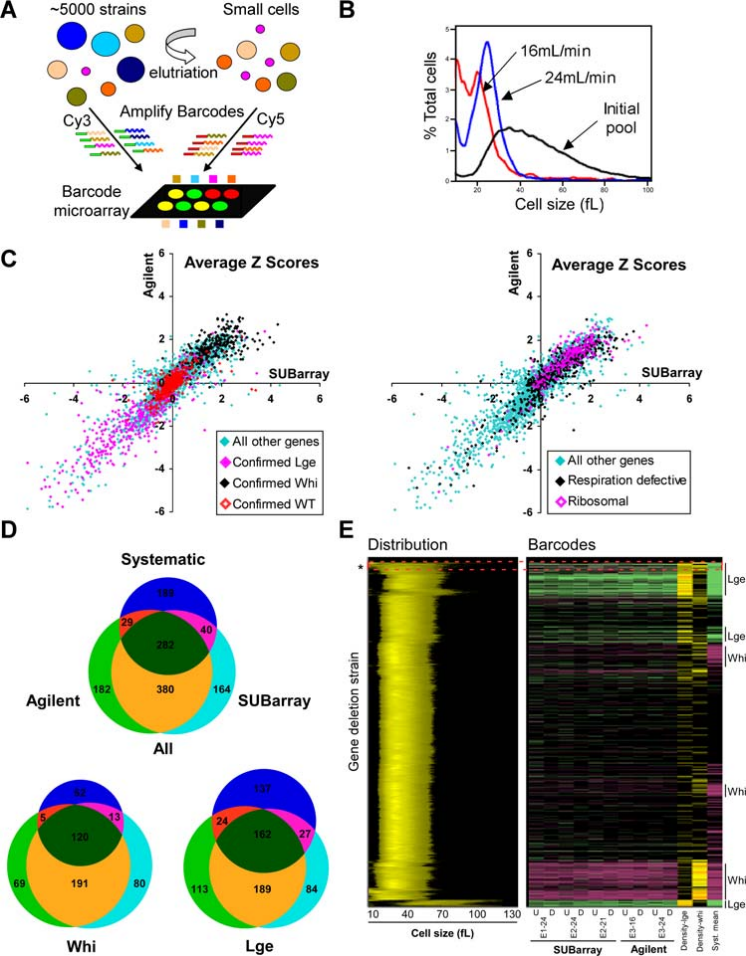
Barcode elutriation cell size experiment. A. Schematic of the experiment. Elutriation of the haploid pool enriches for small cells. Genomic DNA is isolated from cell populations immediately before and immediately after elutriation, differentially labeled with either Cy5 or Cy3, and applied to a barcode microarray. B. Cell size distributions of a characteristic elutriation as determined by use of a Coulter Z2 particle analyzer (Beckman). Increasing the rate of flow through the elutriation rotor increases the median cell size of the elutriated fraction. C. Scatter plot of the average barcode Z score. Agilent (Y axis). SUBarray (X axis). Left. Overlay of barcodes of strains confirmed as either *lge* (black), *whi* (magenta), or wild-type (WT; red) by systematic experiments [28] on all other barcodes (blue). Right. Overlay of barcodes of structural components of the cytosolic or mitochondrial ribosome (magenta) or respiration defective strains [32] (black) on all other barcodes (blue). D. Venn diagrams of overlap between systematic and barcode data using SUBarrays or Agilent arrays. Cell size mutants are as defined in the text. E. Overlap between systematic and barcode data. Clustered cell size distributions are represented by horizontal bars color coded by intensity to reflect the shape of the distributions, with deletion strains on the vertical, and cell size on the horizontal axis. Barcodes, divided into UP (U) and DN (D) tags, with Z scores greater than 1 are represented as Lge (green bars) or Whi (red bars) adjacent to their cognate deletion strain for all experiments (E1, E2, E3) and all elutriation cuts (16, 21, 24 mL/min). SUBarrays and Agilent arrays are shown. The density of *lge* or *whi* barcodes (Z score>1) over a 3 gene window is represented by horizontal bars color coded by intensity (>10% brown; >30% yellow; >70% orange). Only barcodes with significant intensity/SNR were used in density calculations. Gene deletion strains with systematic mean cell sizes >1 SD larger (green) or smaller (red) than average are shown. Systematic *lge* strains with broad distributions and unexpected cell size by barcode are indicated (dotted red box and asterisk).

To prove SUBarrays in a biologically demanding application, we undertook comprehensive analysis of cell size across all viable haploid gene deletion strains. Our previous systematic strain-by-strain analysis of the entire cell size phenome [28] provided a rigorous benchmark with which to assess the SUBarray microarray platform. To measure cell size in parallel on a genome-wide scale, we subjected pools of the haploid deletion collection grown in rich media to centrifugal elutriation. This technique physically separates cells on the basis of size; progressively increasing the flow rate of liquid through a continuous flow rotor in a direction opposite to the centrifugal force expels yeast cells of increasingly larger size from the chamber (Figure 3B). A series of elutriated yeast fractions were harvested and the barcodes derived from cultures obtained immediately before and after elutriation were differentially labeled and hybridized to barcode microarrays (Figure 3A). Deletion strains enriched after elutriation represent small (*whi*) mutants, while those depleted from the small elutriated populations represent large (*lge*) mutants (microarray data are deposited at ArrayExpress; E-MEXP-1200).

To compare the results of the original systematic cell size screen to our elutriation/barcode array experiments, we overlaid our set of known and high confidence *lge* and *whi* deletion strains (mean and median systematic cell size differing by >1 standard deviation [SD] from genome-wide average), as well as our confirmed wild-type deletion strains (mean, median, and mode cell size differing by <0.2 SD from genome-wide average) on a correlation plot of average barcode Z scores using SUBarray versus the Agilent arrays (Figure 3C, left). Approximately 92% of known *whi* strains fell in the upper right quadrant of the plot, i.e., these strains were enriched in both elutriated populations. Correspondingly, 76% of known *lge* strains fell in the lower left quadrant, i.e., these strains were depleted from the elutriated populations. As expected, barcodes that corresponded to strains of wild type size showed neither enrichment nor depletion.

Cell size is a complex biological readout that reflects contributions from cell morphology, bud size and distribution and the balance of growth and division [31]. In particular, any mutation that compromises growth but not division will lead to a small cell size. For example, deletion of genes involved in respiration or in ribosome function confers a small cell size [28], [32]. As expected, respiration defective strains and strains lacking structural components of the mitochondrial and cytosolic ribosome also clustered in the upper right quadrant of the scatter plot that reports small cell size (Figure 3C, right).

To compare the individual cell size deletion strains identified by each method, we first defined two-step filters for significant enrichment or depletion by barcode. In the first step, Z scores for each array (typically in the range of 1.0–1.3) were chosen to exclude >95% of the high confidence wild-type gene set. In the second step, we filtered putative *whi* or *lge* gene deletions according to the total number of times a replicate for either barcode tag was detected above the array-specific Z score thresholds. Using ROC curves, we again found that incorporating data from both tags and from all replicate spots gave the best results (Figure S3A). We further adjusted the threshold of the second step filter to limit dye-swap artifacts. A detailed description of this analysis is provided in the Supplementary material (Supplemental Text S1 and Supplemental Charts S1). From ROC plots, we found that SUBarrays and Agilent arrays were comparable in their ability to distinguish size mutants from wild-type populations (Figure S3B), though we did observe a larger number of high intensity dye swap artifacts with Agilent arrays (Figure S3C,D).

Despite employing entirely different measures of cell size over hundreds of size mutants, i.e., size profiles of individual cultures measured by electrolyte displacement on a Coulter channelizer versus a population continuum of physical sizes separated by centrifugal elutriation, we observed a very substantial overlap between the high confidence systematic data and both sets of barcode data: ∼52% of systematic phenotypes were reported in both sets of elutriation/barcode data and ∼65% in at least one set. This is comparable to overlap of 60–90% previously reported between barcode analyses and other systematic phenotypic datasets, which typically cover 5- to 10-fold fewer mutant genes [30], [32]–[35]. Of the 189 cell size mutants identified only in the systematic data, 38 (∼20%) had insufficient signals for both barcode tags, and 96 (∼52%) showed the expected enrichment or depletion by barcode (mean and median Z score for either platform greater or less than zero, respectively), but were below the filtering thresholds employed. An even greater overlap in phenotypic profiles was recovered between the two different barcode array formats (∼76% overlap; Figure 3D). 57% of deletion strains with cell size phenotypes detected by both elutriation/barcode array formats were absent from the high confidence systematic data set. These strains have moderate systematic size phenotypes, with average mean cell sizes 0.25 SD smaller and 0.3 SD larger than the population average, and thus represent *bona fide whi* and *lge* mutants, respectively (not shown).

We noted substantially greater overlap between the three methods for *whi* mutant strains as compared to *lge* mutants. This effect is likely due to the difference in the methods of cell size determination. Because elutriation selects for the smallest cells in the population, it is thus biased towards the positive identification of *whi* strains, whereas direct size analysis based on both mean and median of the size distribution is not biased in this fashion. Moreover, a *lge* deletion strain with a broad size distribution that includes small G1 daughter cells, even if most cells are large, would not be classified in the same manner by elutriation and systematic size analysis. This trend can be observed when the systematic cell size data is displayed as a heat map of cell size distributions and compared to *lge* and *whi* strains identified by barcode experiments (Figure 3E). While *whi* strains identified systematically (i.e., shifted left on the distribution; red bars in Syst. Mean column) consistently co-occurred with a high density of enriched barcodes (red bars), the *lge* phenotype was less consistent. In most cases, *lge* strains identified systematically (i.e., shifted right on the distribution; green bars in Syst. Mean column) co-occurred with a high density of depleted barcodes (green bars). However, in the case of the systematically defined *lge* deletion strains with broad distributions (boxed and marked with an aster), the density of *lge* barcode mutants detected was much lower, and many strains were actually detected as *whi*. Aside from this difference imparted by the method of enrichment, the barcode experiments appear remarkably consistent with systematic size determinations given the differences in methodology. The complete list of significant barcode values for Agilent and SUBarray replicate arrays is provided in the Supplementary materials (Data S1) as is supporting data for Figure 3E (Data S2).

**Figure 4:**
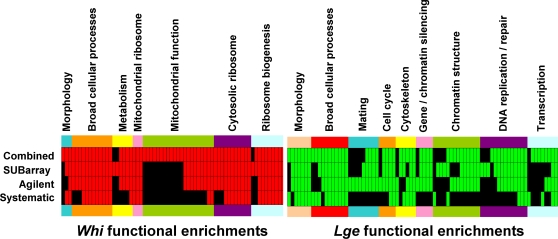
Barcode elutriation and systematic cell size enrichment of gene ontology (GO) annotations. Statistical enrichment from the haploid deletion set was calculated using cumulative hypergeometric probability functions (CDFs) with Bonferroni correction for either the high confidence systematic cell size data; SUBarray or Agilent array data; or the combined set of all *whi* or *lge* deletion strains identified. Significant enrichment for *whi* (red bars) or *lge* (green bars) strains is defined by [p*N<0.01, where N is the number of categories per subset)]. GO component (N = 330); GO process (N = 1380); Morphology (N = 11) [27]. GO categories are arranged by related function.

The consistency between methods is also evident across Gene Ontology (GO; http://www.geneontology.org) component and process annotations (Figure 4). *Whi* genes were enriched (p<0.01) for GO functions in growth related processes, including cytosolic or mitochondrial ribosome assembly and function, mitochondrial function, and metabolism. *Lge* genes were enriched (p<0.01) for GO functions in cell cycle processes, including DNA replication and repair, chromatin structure, and the cytoskeleton. These observations are readily explained by shifts in the balance between growth and division: a decrease in growth rate without a compensating decrease in cell division rate leads to smaller size, whereas a converse delay in division in the absence of any attenuation in growth rate leads to larger size. Despite the large number of additional mutants recovered in the elutriation/barcode experiments, most GO categories were proportionately represented in each dataset.

### Atomic force microscopy (AFM) images of spotted probes

**Figure 5:**
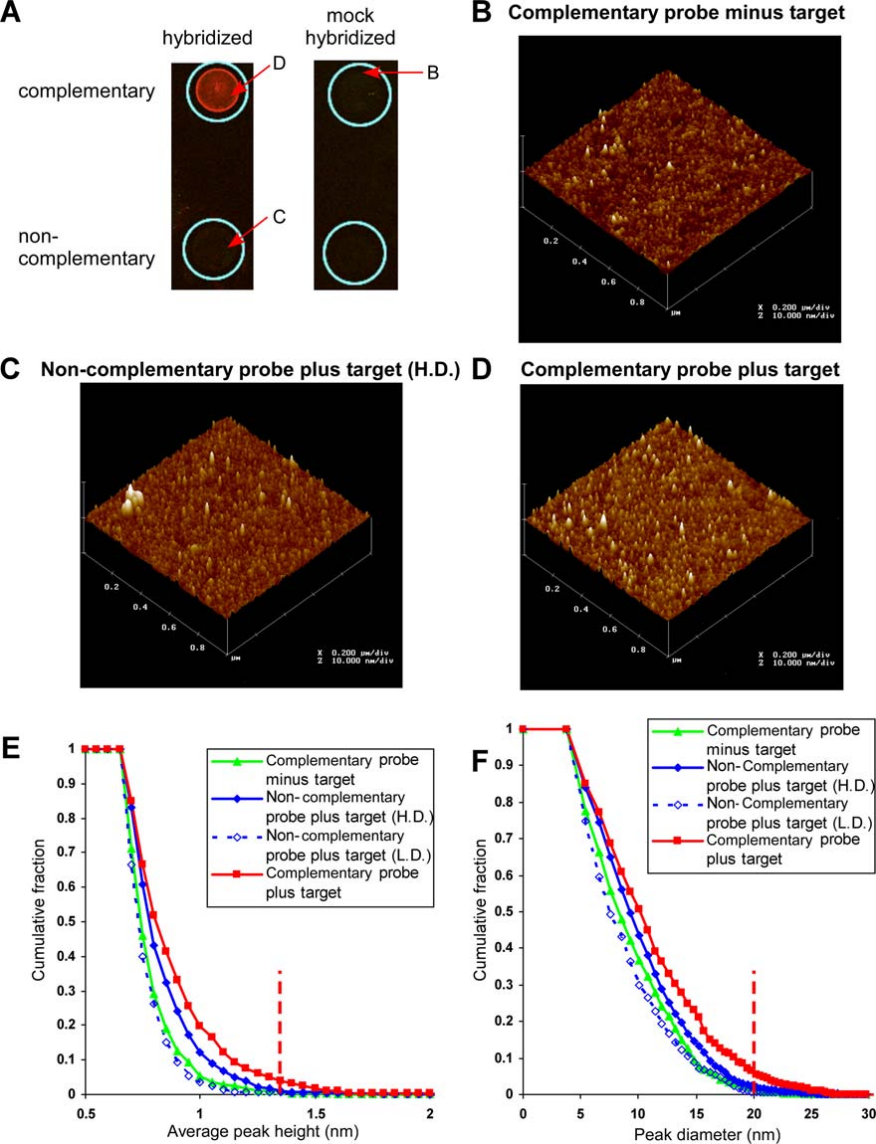
Atomic force microscopy (AFM) of hybridized and mock hybridized spotted 20mer barcodes. A. Fluorescent imaging of hybridized and mock hybridized slides. Each slide contained two spots of oligonucleotides complementary or non-complementary to the labeled target sequence. The location of the AFM scans is indicated by a red arrow. B. Mock hybridized barcode oligonucleotide. 1 µm×1 µm scan of the surface arrangement. Pixels are ∼2 nm on the x and y axis, and <100 pm on the z axis. C. High density non-complementary barcode oligonucleotide hybridized in the presence of its non-cognate target sequence. The location of a region of aberrant signal omitted from the analysis is indicated by a red arrow. D. Complementary barcode oligonucleotide hybridized in the presence of its cognate target sequence. E. Cumulative frequency of peak number with increasing height cut-off for high (solid blue) and low density (dashed blue) non-complementary and complementary barcode oligonucleotides minus (green) or plus (red) hybridized target. F. Cumulative frequency of peak number with increasing diameter cut-off for high density (solid blue) and low density (dashed blue) non-complementary and complementary barcode oligonucleotides minus (green) or plus (red) hybridized target. The method of estimation of peak dimensions is described in the Supplementary material (Supplemental text S1). The height and diameter at which 99% of mock hybridized peaks are excluded is indicated by a red dotted line.

Given that short oligonucleotides are typically linked covalently to the glass surface of a slide, and as previous studies have shown loss of significant specific hybridization with less than 13 contiguous bases of homology [36], we wondered how 20 mers on the surface (hereafter probe) were able to specifically hybridize with labeled barcode amplicons (hereafter target). To address this, we used AFM to image the arrangement of both complementary and non-complementary spotted probe sequences, with or without hybridization to a labeled target sequence, at the single molecule level (Figure 5A). AFM affords a 2 nano meter resolution on the horizontal axis and a better than 1 Angstrom (or 100 pico meter) resolution in the vertical axis [37]. We scanned hybridized or mock-hybridized arrays for fluorescence prior to AFM analysis in order to choose an area of maximum hybridization. As expected, signal was significantly above background only in the presence of a complementary pair of target and probe sequences (Figure 5A; arrows mark the 1 µm×1 µm areas chosen for AFM scans).

When we examined a complementary probe-containing region, mock hybridized in the absence of target barcodes (Figure 5B), we observed peaks with mean diameters of approximately 8 nm and heights of 0.7 nm (Table 2). These results are similar to the arrangement and measurements previously reported for spotted single-stranded 50mer and 25mer DNA oligonucleotides [38]. The characteristics of a low density (L.D.) region of spotted probe hybridized against a non-complementary target sequence were similar (Table 2; not shown). A high density (H.D.) region within the same probe spot also appeared similar (Figure 5C), though with a modest increase in mean peak heights and diameters (Table 2).

**Table 2:** Peak attributes of atomic force microscopy measurements.

Peak base	*Complementary minus target*	*Non-complementary*		*Complementary plus target*	*Hansma et al. (25mer* 1 *; 50mer)*
		*L.D.*	*H.D.*		
Mean diameter (nm)	8.4	7.9	9.4	10.4	14^1^; 11
Median diameter (nm)	7.8	6.8	8.7	9.8	
95% inclusion limit (nm)	16.6	17.0	17.9	21.0	
**Peak height**					
Mean height (nm)	0.73	0.71	0.78	0.82	1; 1
Median height (nm)	0.69	0.67	0.73	0.76	
95% inclusion limit (nm)	0.95	0.90	1.10	1.25	
**Number of peaks**	553	517	1036	767	
**Estimated bound peaks (% bound)**	3 (0.58%)	3 (0.54%)	8 (0.77%)	24 (3.1%)	

The attributes of the peaks were calculated according the methods described in the Supplementary material (Supplemental text S1). The 95% inclusion limit is the point at which 95% of the peaks have heights or diameters less than the indicated value. Bound peaks are defined as those with heights and diameters greater than 99% of the complementary probe minus target condition (height>1.3 nm; diameter>20.2 nm).

1 In this study [38], the 25 mer population appeared non-homogenous and was likely composed of probe aggregates, leading to a larger diameter relative to the 50 mer.

When we scanned a probe region hybridized in the presence of its complementary target sequence (Figure 5D), there was a marked increase in the number of larger height pixels relative to controls. However, despite an increase in peak dimensions, the majority of peaks still displayed similar properties to the mock-hybridized region, as observed in cumulative frequency plots for peak height (Figure 5E) and diameter (Figure 5F). This result implies that a large portion of the spotted probe sequence remained unbound by target. Peak diameter appeared to be the defining characteristic of hybridized regions: for thresholds that excluded 95% of peaks in each condition, all negative conditions displayed diameters within 2 nm of one another (16.6–17.9 nm), whereas hybridized complementary regions had a much larger threshold, at 21.0nm (Table 2, Figure S7). Applying an exclusion limit of 99% of the mock-hybridized condition (>1.3 nm average height and >20.2 nm diameter), which was chosen because it was the only condition where there was no possibility of target-probe hybridization, we estimated the percentage of bound probe at 3.1% in the hybridized condition versus <0.77% in the negative control conditions (Table 2). We suspect that background signal in the absence of specific hybridization was in fact far lower than estimated, as when we examined defined probe-target peaks in the high density spotted probe regions, we found a high incidence of shouldered peaks that likely result from juxtaposed spotted oligonucleotide (Figure S8).

To determine if our estimates of hybridized probe-target peaks were consistent with the observed fluorescent signals in the region scanned by AFM, we spotted a range of known concentrations of the Cy5-labeled primer used in target amplification on GAPS™II slides. From this, we generated a standard curve relating fluorescent signal and target molecule number per 1 µm^2^ (Figure S9). Given the location of the 1 µm×1 µm AFM scan, we defined a range of possible signal intensities for this region, from 3000–6000 under the scan settings used. This corresponds to an expected 6-30 target molecules per 1 µm^2^, consistent with the 24 probe-target peaks estimated by AFM (Table 2, Figure S9).

## Discussion

Terminal modification of short oligonucleotide probes for covalent coupling to slide surfaces has long been the accepted standard for fabrication of short oligonucleotide arrays [39]. Yet surprisingly, we found that the absence of a 5′-amino linker did not overtly affect hybridization performance. Comparisons of unmodified and amino-linker containing probes, even on an uncharged and non-optimal surface for spotting of unmodified probes, SuperAldehyde®, showed only a moderate defect in performance. This defect was noted using previously published hybridization conditions [30], which we have improved substantially for our arrays. While published hybridization conditions were sufficient for our pilot study, application of our modified protocol is essential for optimal SUBarray performance. As a caveat to our approach, while these conditions did not negatively impact on barcode microarray specificity, the suitability of this approach to other microarray applications utilizing longer DNA or RNA targets would need to be assessed.

In the context of a genome-wide experiment, SUBarrays showed comprehensive performance that was on par with Agilent covalently-linked 20mer arrays. Further, results from SUBarrays were sufficiently reproducible to ensure biological accuracy, though, likely due to higher background and the use of background subtracted intensities, displayed less precision than the Agilent platform, particularly for higher Z score values, where one signal was close to background (Supplementary text S1, Figure S4, S5). The cell size distribution across the haploid deletion pool represents a robust test case for barcode array analysis, as the results are largely invariant, even when one compares different elutriations performed on different days, and different elutriation fractions within the same experiment. Even though our comparison with arrays from Agilent was based on entirely different elutriations, we obtained a high degree of correlation between these two platforms (r = 0.92). Interestingly, we observed an increased frequency of dye-swap artifacts in the Agilent array experiments, even at high signal intensity and high log ratios, which were not observed with our SUBarray platform. These artifacts occurred in a common set of barcodes in two independent dye-swap experiments using different biological samples. Whether this observation represents a defect in a small subset of arrays, an issue with sub-ideal hybridization conditions, or a general trend in similar arrays, cannot be assessed without further analysis.

Because we analyzed the haploid deletion set, which excludes a significant fraction of barcodes from essential genes, we could define a large set of both false and true barcodes, thereby allowing us to optimize our filtering strategy. We found that a two step filtering approach could be applied to different biological replicates, and even different types of experiments (not shown), as long as the pool contained the same cohort of deletion strains. We found this method to be much better at reducing the number of false positives than a simple static intensity or SNR threshold. Similarly, by exploiting deletion strains known to possess wild-type characteristics during our size selection experiment, it allowed us to define appropriate Z score thresholds on the basis of exclusion of these strains from the list of cell size mutants. This method of excluding false negative signals is a viable alternative to applying static thresholds, trying to maximize a small number of true positive hits, or applying mixed variance models that derive their power from extensive replication of both control and experimental samples. Our strategy of false negative exclusion is particularly compatible with the use of strain or target/barcode controls that are spiked-in at known ratios prior to amplification or hybridization [40], [41].

We applied the SUBarray platform to the problem of cell size control, and demonstrated a very substantial overlap between systematically determined sizes [28] and those obtained by barcode, as well as a strong concordance in the types of genes isolated by each method. Since the systematic strain-by-strain approach to cell size determination on a genome-wide scale is very time consuming, initial studies of the cell size phenome were very limited in the number of conditions tested [28], [42]. Population level analysis by barcode array profile enables many different conditions to be surveyed in rapid succession. We are currently applying the barcode approach to interrogation of cell size phenotypes in the context of different nutrient sources, ploidy conditions, genetic backgrounds and in the presence of various chemical reagents as well as ‘perturbagens’ to systematically decipher the global network that coordinates cell growth and division.

An unexpected feature of the unmodified 20mer oligonucleotides is the ability to specifically hybridize to complementary sequences even after UV cross-linking to the slide surface. The UV cross-link is thought to occur predominantly through free radical generation on thymine bases and subsequent covalent bond formation [16], [17]. If significant hybridization requires at least 13 contiguous bases of homology [36], then one would predict an enrichment of thymidine-containing sequences in non-functional barcodes. However, we observed no significant difference in thymidine content between functional and non-functional barcodes, across the sequence, or at any internal position (not shown), similar to previous reports [26].

While it is possible that hybridization occurs cooperatively in large DNA clusters, since the DNA peaks we observed by AFM are of comparable size to those of single stranded 25mer and 50mer sequences observed in previous AFM studies [38], it seems unlikely that each peak contains more than one target or probe sequence. Moreover, since each peak in the complementary hybridized condition is separated by an average of 35–40 nm (and each peak has an average diameter of 8 nm) compared to a peak diameter of ∼20–25 nm in the case of bound probe, interactions between a single target molecule and multiple spotted probes would seem physically impossible. Further, our predictions of 6-30 bound target molecules based on correlation of fluorescent signals and target number closely match our estimates of bound peaks in our hybridized condition (24), suggesting that a single target sequence binds to each probe peak. That the peak diameter increases by two-fold upon hybridization is unsurprising given that the PCR products include 30 bases of primer sequence in addition to the barcode complement. However, while the height of each unhybridized peak is consistent with a single strand/base of DNA extending from the surface (0.7–0.8 nm), the peak diameter is larger than would hypothetically be required to contain a compact 20mer oligonucleotide with base-stacking interactions (7–9 nm observed versus 3–4 nm length predicted for an extended oligonucleotide). Similarly, hybridization results in a doubling of the diameter of each peak, which is more than would be necessary to contain a single probe and target molecule. This may be an issue of over-estimation of the peak diameters due to the insufficient resolution of AFM. However, we cannot eliminate the possibility that each peak contains more than one probe, and that either hybridization occurs through partial hybrids between a single target and multiple probes, or that multiple probe-target interactions occur within each peak.

It could be that a substantial proportion of the spotted oligonucleotide is non-functional due to thymidine cross-linking, especially as only an estimated ∼3% binding of target to the probe was observed by AFM. However, if the probe densities achieved on our microarrays are similar to those observed by AFM, based on our correlations of fluorescent intensity and target molecule number, we estimate that the most intense spots on our array achieve near saturation of target-probe binding, suggesting that the majority of spotted probe is functional.

In summary, arrays of unmodified 20mer oligonucleotide barcode arrays exhibit specific and reproducible hybridization behavior that enables the systematic dissection of complex phenotypes such as cell size. Given the excessively greater cost of modifying oligonucleotides with reactive linkers, and the relative time and cost of ink-jet synthesis, our results document a cost-saving alternative in array fabrication. The substantial savings in initial array construction are likely to be especially advantageous for microarray applications in which oligonucleotide quality control is the primary consideration. Given current constraints in single molecule detection [43], which can only be achieved under low density conditions, unmodified 20mer oligonucleotide arrays may also enable cost effective surveys of weakly expressed genes.

## Materials and Methods

### Microarray fabrication

Barcode probe sequences were as published [27], [29]. Array design and probe sequence information of the 13K v.2 Universal Barcode has been submitted to the European Bioinformatics Institute (EBI); array information can be accessed at ArrayExpress (E-MEXP-1200). Pilot barcode modified or unmodified 20 mer oligonucleotides were constructed according to standard 25 nmol scale protocols on a PolyPlex 96-well Oligonucleotide Synthesizer (GeneMachines/Genomic Solutions, Ann Arbor, MI). 5′-amino modification with C3 and C6 linker was used in synthesis of UP and DN barcodes, respectively. Probes used in pair-wise comparisons consist of the UP and DN barcodes for YBL090W, YBL091C, YBL093C, and YBL094C. Sequences are included in a GeneList file in the Supplemental material (List Data S1). Probes were characterized using mass spectrometry to ensure correct sequence and incorporation of the modified linker. Except for in-house oligonucleotides used in the pilot experiments, all oligonucleotides were synthesized by Illumina Inc (San Diego, CA).

Prior to printing, oligonucleotides were dissolved in Micro Spotting Solution Plus printing buffer (Telechem, Sunnyvale, CA) at 40 µM. Pilot microarrays were printed in quadruplet on all slides using BioRad VersArray ChipWriter Pro and SMP3 stealth pins (Telechem). Oligonucleotides spotted on SuperAldehyde® (Telechem) or GAPS™II (Corning, Corning, NY) were UV cross-linked at 200 mJ/cm^2^. Pilot arrays were sequentially washed twice in 0.1% SDS, three times in double deionized water (ddH_2_O, Millipore UltraPure), in boiling ddH_2_O for 2–3 minutes; and were dipped 5 times in 95% ethanol and spun dry. Arrays were stored in vacuum desiccators before use. The post–printing processing procedure for 13K feature barcode arrays on GAPS™II slides was carried out as described above, with the exception that slides were first immersed and washed in 1% (w/v) BSA (fraction V), 0.1% SDS, 3× SSC for 2 minutes with mild agitation. Quality control of printed DNA was performed by hybridizing arrays with a 7.5 µM Cy3-labeled random 9-mer (Operon, Huntsville, AL) in Hybridization Solution [4× SSC, 1 mg/ml poly-dA, 50 mM HEPES pH 7 (or Tris pH 7.5), 0.2% SDS]. Prior to hybridization, Cy3 9mer hybridization mix was heated to 85°C, cooled, loaded between the array surface and a LifterSlip (Erie Scientific, Portsmouth, NH), incubated at 25°C for 3–5 minutes, and washed sequentially in 2× SSC, 0.2% SDS; 2× SSC; and 0.2× SSC before spinning dry.

### Agilent covalently coupled barcode microarrays

“Agilent Custom Yeast Barcode, Version 1.0” (ArrayExpress accession: A-MEXP-842) was designed by Tim Hughes (Centre for Cellular and Biomolecular Research, University of Toronto) and was a gift from Charlie Boone (Centre for Cellular and Biomolecular Research, University of Toronto). This array was custom-made using Agilent proprietary inkjet technology and consists of one grid of 215×105 features. The array consists of 22575 features made up of 4771 unique UP and 4609 unique DOWN tag pairs in duplicate and triplicate for all non-essential yeast deletion strains according to Giaever et al. [27] and Agilent proprietary positive and negative controls.

Essentially the Agilent Custom Yeast Barcode arrays differ from the in-house “SLRI_Yeast_Barcode_13k, Version 2.0” arrays by probe sequences and their surface substrates. The former consists of barcode probes with an additional stretch of 10 of T's (served as a spacer) at the 3′ end of the *in situ* synthesized covalently linked probes.

### Growth and cell-size selection

Haploid yeast deletion strains from the *MAT* ***a*** deletion collection were grown as individual colonies on XY glucose solid medium (YEPD+100 mg/L adenine+200 mg/L tryptophan) containing 200 µg/ml G418, pooled, aliquoted, and frozen in XY containing 15% glycerol. This pool was used to inoculate all elutriation experiments. Approximately 1.5×10^7^ cells from the pool were used to inoculate each of two 1L cultures in XY+2% glucose containing 100 µg/ml G418 for cell-size selection experiments. Log phase cultures were harvested at a cell density of 1-3×10^7^ cells/mL and were loaded into a 40 mL JE-5.0 elutriation rotor in a J6-Mi centrifuge (Beckman, Fullerton, CA) at 16°C. Successive elutriation size fractions were obtained at the indicated flow rates and at a rotor speed of 2400 rpm. Genomic DNA was extracted from elutriated fractions and from samples of the pool culture taken immediately before elutriation.

### Barcode amplification, hybridization, and image analysis

UP and DN universal barcode tags were amplified and fluorescently labeled in PCR reactions using the UP-tag [Primers U1 (5′-GATGTCCACGAGGTCTCT) and U2-Cy3 (5′-Cy3-GTCGACCTGCAGCGTACG)] and DOWN-tag [Primers D1 (5′-CGGTGTCGGTCTCGTAG) and D2-Cy3 (5′-Cy3-CGAGCTCGAATTCATCGAT)] for the control and UP-tag [Primers U1 and U2-Cy5 (5′-Cy5-GTCGACCTGCAGCGTACG)] and DOWN-tag [Primers D1 and D2-Cy5 (5′-Cy5-CGAGCTCGAATTCATCGAT)] for the experimental samples. Dye-swap experiments were performed with reciprocal labeling. All amplification primers were from Operon.

Briefly, 50 µL PCR reaction mixtures containing 1.5 mM MgCl_2_, 0.2 mM dNTPs, 100 ng yeast genomic DNA, 1 µM of the primer pair and 5 units of Taq polymerase (Invitrogen, Burlington, Ontario) was brought to 94°C for 3 minutes and subjected to 38 cycles [94°C, 30 s; 50°C, 30 s; 72°C, 30 s] and terminated at 72°C for 5 min. PCR reaction products were ethanol precipitated in 0.3 M sodium acetate (pH 5.2) in the presence of 5 µg of linear acrylamide (Ambion, Austin, TX) and a ten fold excess of blocking primers (U1, D1, U2block: 5′-CGTACGCTGCAGGTCGAC, D2block: 5′-ATCGATGAATTCGAGCTCG). Precipitated PCR products were dissolved in 5 µl of ddH_2_O and mixed into 60 µL of DIG Easy Hybridization (Roche, Laval, Quebec) solution. Hybridization targets were heated to 95°C, quick chilled on ice, and kept at 50°C covered from light until applied to arrays. Hybridization was performed at 25°C overnight (>12 hours) under a LifterSlip (Erie Scientific). Hybridized arrays were washed sequentially at 30°C with 6× SSPE, 0.05% Triton X-100; 25°C with 2× SSPE, 0.05% Triton X-100; and 0.2× SSPE, 0.05% Triton X-100; and 0.2× SSPE before spinning dry. Hybridized unmodified oligonucleotide pilot and 13K arrays were imaged using a GenePix 4000B Array Scanner (Axon Instruments/Molecular Devices, Sunnyvale, CA). Samples for Agilent arrays and for the pilot barcode array were prepared and hybridized as described previously [30]. Briefly, PCR was performed with Platinum PCR SuperMix (Invitrogen) as above for 35 cycles. PCR probes were heat denatured at 100°C for 1 minute. Hybridization was performed in 3.5 mL of 1× SSTE (1 M NaCl, 10 mM Tris.Cl, pH 7.5, 0.5% Triton X-100), containing 70 µl each of Cy3 and Cy5 labeled probes, and 1.3 µM of each blocking primer. Hybridization was performed at 40°C for 3 hours in a rotator hybridization oven in a heat sealable bag (Kapak Corporation, Minneapolis, MN). Hybridized arrays were washed sequentially at 42°C with 6× SSPE, 0.005% Triton X-100; 25°C with 2× SSPE, 0.005% Triton X-100; and 0.2× SSPE, 0.005% Triton X-100; and 0.2× SSPE before spinning dry. Agilent microarrays were scanned with a GSI Lumonics machine (Moorpark, CA). Initial scanning was used to assess print quality. All images were processed with GenePix Pro v.6 (Axon Instruments/Molecular Devices). Data were LOWESS normalized using Vector Xpression 3 (Invitrogen), and were subsequently analyzed in Excel (Microsoft, Redmond, WA) as described in the Supplementary material (Supplemental text S1).

### Atomic Force Microscopy (AFM)

Complementary and non-complementary oligonucleotide probe sequences were 5′-TACTGAGCGGCATGTCACTG (WHI5/YOR083W-UP) and 5′-CCAGTTCGGGAATGTGCTTC (MBP1/YDL056W-UP). Probes were brought to 40 µM in 10 µl of Micro Spotting Solution Plus (Telechem), spotted on GAPS™II slides, air dried, and hybridized as above for 13K feature unmodified oligonucleotide arrays, with the exception that precipitation was performed in the absence of linear acrylamide and blocking primers. The Cy5 labeled targets (5′-Cy5-GTCGACCTGCAGCGTACG-CAGTGACATGCCGCTCAGTA-AGAGACCTCGTGGACATC-3′) were generated by a PCR reaction using genomic DNA from the yeast deletion strain *whi5/yor083wΔ*. AFM experiments were carried out on a Digital Instruments Dimension 3000 (Model MPP-11100) in tapping mode, using the etched *Si* probes with tip diameter at 17 nm, and pyramidal shape and front, back, and both side angles of 15°, 25° and 17.5°, respectively (RTESP, NanoDevices/Veeco Probes, Camarillo, CA). Voltage output files were first processed with the software NanoScope III for initial roughness, grain size, and density analyses. Subsequent statistics of the peak heights and peak areas of hybridized and non-hybridized DNA oligonucleotide probes on the GAPS™II slides were calculated using Excel (Microsoft) and according to the header information of the voltage output files. The voltage to height (in nanometer) conversion was: HEIGHT = (Data_point * Full_data_range)/(2ˆ(8*Bytes/Pixel)), where Full_data_range = Z_scale * Z_scan_sensitivity. A custom PERL script was used to adjust local background height. Three-dimensional rendering and color surface presentations of AFM results (presented in the Supplemental Figure S8) were produced in MATLAB (Version R2006a, MathWorks, Natick, MA). A complete description of the analysis is provided in the Supplemental material (Supplemental text S1, Figure S10).

### Fluorescence calibration curves

The concentration and percent Cy5-incorporation of each of two aliquots of the primer U2-Cy5 (5′-Cy5-GTCGACCTGCAGCGTACG) used in amplification of the target barcode for AFM were determined by absorbance at 260 nm and 650 nm, respectively. These primers were each used to create 5-fold dilution series in ddH_2_0. 1 µL of each dilution was spotted on a GAPS™II slide. Independent dilution series were generated and spotted on a duplicate slide. Both slides were scanned with comparable settings to the slides used in AFM.

### URLs

http://www.mshri.on.ca/microarray/ and http://www.mshri.on.ca/tyers/

## Supporting Information

Supplemental Text S1 Detailed description of a) supplementary scatter plots of SUBarray and Agilent replicate arrays, and the methods for analysis of b) barcode microarray data and c) atomic force microscopy data are included in this supplemental text.
(0.06 MB DOC)

Figure S1 Analysis of previously defined anomalous barcodes [1]. A. The log2 ratio of the Cy5/Cy3 (M) channels is plotted versus the average log2 value of the signal intensity in each channel (A) for 8 replicates of each barcode. Barcodes from sub-populations of non-essential deletion strains from chromosome 2 are labeled with Cy5 (Chr2_1; black) and Cy3 (Chr2_2; magenta). Negative control sequences are shown (red). Previously defined anomalous barcodes are plotted in gold. B. Frequency of the average log2 value of the signal intensity from each channel for anomalous and all other barcodes. Barcode values are an average of all replicates.
(0.89 MB TIF)

Figure S2 Use of ROC curves in defining intensity thresholds. A. True and false barcodes or genes are defined based on their presence or absence from the experimental pools, respectively. The 45{degree sign} tangent (dashed black) to the characteristic ROC plots (red, blue) is the point at which the rate of loss of false positives equals the rate of loss of true positives. B. Comparison of SNR thresholds defined by different measures of microarray data quality. Dotted line indicates the maximum obtained using true positive and false positive data (green) to set thresholds. Plots obtained from the measures of the average standard deviation (STDEV) between analogous spots on different arrays (blue) or the Pearson's correlation coefficient between arrays (red) begin to plateau at a similar threshold. A representative comparison between two dye-swap replicates is shown for all methods. C. Filtering according to the total number of barcodes with significant signal across multiple arrays (solid), rather than by the SNR from individual arrays (dashed) increases the ability to distinguish false and true data. Data is plotted for UP tags, but DN tag ROC plots are analogous. D. Filtering according to the total number of barcodes with significant data (Total; blue) yields slightly better data than using the fraction of total barcodes (% Total; green) or the maximum number of significant replicates for the best of the UP or DN barcode only (Max; red), or either the UP (black) or DN (black dashed line) tag data alone.
(1.49 MB TIF)

Figure S3 Use of ROC plots in defining Z score thresholds for significant hits. A. True positive (either *whi* or *lge*) and false positive (wild-type) genes are defined based on systematically confirmed size characteristics. Individual array Z score thresholds (dashed black) are defined at the point that removes at least 95% of the false positive genes. Filtering by the total number of significant values across all arrays for both barcodes (green) yields better data than the fraction of possible hits (red), or the average (blue) or maximum (dashed black line) Z score for both UP (black) or DN (not shown) barcodes. ROC plots are shown for *whi* strains only. Data are analogous for *lge* strains. B. Comparison of the performance of optimized filtered data from Agilent (blue) or SUBarrays (red) for both *whi* (solid) and *lge* (dashed) strains. C. Dye-swap analysis from Agilent (left) and SUBarrays (right). Dye swap and technical replicate spots with consistent enrichment or depletion by elutriation (magenta) or those with any inconsistent values (blue) are plotted on a graph of the average absolute value of the log2 ratios (M) versus the average log2 value of the signal intensities (A). Barcodes with high intensities and high log ratios are the most consistent. Inconsistent high intensity, high log ratio barcode replicates represent dye swap artifacts and are more frequent in these Agilent arrays. Comparisons of Agilent and SUBarrays are between two and four replicate experiments, respectively. Consistent values agree for all experiments.
(2.60 MB TIF)

Figure S4 (A–D) Scatter plots for the best (left) and worst (right) correlated SUBarray replicate arrays (as defined by Pearson's correlation coefficients) for technical (A), dye-swap (B), and intra- (C) or inter-experimental (D) replicate arrays. Z scores (as defined in the text) from on-chip replicate spots were averaged prior to generation of the scatter plot. Each experiment represents an independent set of PCR reactions. Dye-swap Z score values were multiplied by a factor of -1. All array data are derived from a log2 ratio of elutriated/pre-elutriated samples. Arrays are defined as: 592 (E2-21ml/min, log2[Cy5/Cy3]); 593 (E2-24ml/min, log2[Cy5/Cy3]); *597 (E2-21ml/min, log2[Cy3/Cy5])*; 806 (E2-21ml/min, log2[Cy5/Cy3]); *807 (E2-21ml/min, log2[Cy3/Cy5])*; 808 (E1-24ml/min, log2[Cy5/Cy3]). Dye-swap replicates are italicized.
(2.03 MB TIF)

Figure S5 (A–B) Scatter plots for the best (left) and worst (right) correlated Agilent replicate arrays (as defined by Pearson's correlation coefficients) for dye-swap (A) and intra-experimental (B) replicate arrays. An intra-experimental comparison between two arrays with the same labeling scheme is indicated by an asterisk and represents an estimate of technical replication; the average Pearson's correlation coefficient is listed in Table 1 (Average r = 0.95). Comparisons were executed as in Figure S4. Arrays are defined as: FR16 (E3-16ml/min, log2[Cy5/Cy3]); *FF16 (E3-16ml/min, log2[Cy3/Cy5])*; FR24 (E3-24ml/min, log2[Cy5/Cy3]); *FF24 (E3-24ml/min, log2[Cy3/Cy5])*. Dye-swap replicates are italicized.
(1.38 MB TIF)

Figure S6 Scatter plot of the average Z scores from Agilent (y-axis) and SUBarrays (x-axis). Z scores were averaged from all arrays (Agilent, n = 4; SUBarray, n = 6). Comparisons were executed as in Figure S4.
(0.12 MB TIF)

Figure S7 Scatter plot of the dimensions of all peaks (squares) identified by atomic force microscopy. 95% exclusion limits for each scan area are shown as colored diamonds. Peaks from a region lacking any probe (yellow) are less numerous (n = 103) and have significantly different properties than all other peaks.
(0.16 MB TIF)

Figure S8 Background adjusted plots of the AFM scan regions. Data from original scans was adjusted to reduce background variation in the height of the glass surface. Peak height for each region is color coded to height. Both complementary probe minus target (A) and low density non-complementary probe plus target (B) show few large height peaks, while high density non-complementary probe plus target (C) and complementary probe plus target (D) show many more. However, the majority of the large height peaks in the high density non-complementary condition appear to have shoulders (E, white arrows), while the those in the complementary condition do not (F), suggesting that these peaks may be an artifact of high density, and represent two juxtaposed peaks. The region boxed in E has very different surface arrangement than any other peaks observed, is marked in Figure 4C as aberrant, and was omitted in all analyses.
(7.58 MB TIF)

Figure S9 Fluorescence calibration curve for Cy5-target abundance. Replicate slides with known dilution series of one of two Cy5-labelled UP primers were scanned under comparable PMT and power settings to the AFM run. Mean signal intensities for each spot were plotted versus the total number of target molecules per total spot area (μm^2^). The potential range of signal intensities (3000–6000) for the scanned AFM region was used to estimate the theoretical target concentration (6-30; equivalent to hybridized probe-target peak number) in the scanned AFM region.
(0.10 MB TIF)

Figure S10 Pixel and adjusted analysis of AFM data. A. Pixel distribution for a scanned region containing no probe (red). Shown for comparison is the left side of this distribution reflected across its modal point (blue dashed) B. Residual distribution of scan regions after removal of reflected background distributions. To remove an aberrant region containing abnormal peaks, histogram represents only half of the high density non-complementary probe containing region. C. Vertical strip of AFM scan before (blue) and after (green) removal of estimated background (brown). D. Pixel distribution before (red) and after (blue) background correction.
(1.53 MB TIF)

Table S1 Breakdown of microarray construction cost by item.
(0.12 MB TIF)

Supplemental Charts S1 Flow charts describe the algorithms for 2-step filtering of ‘Intensity’(to generate present and absent calls) and ‘Z score’(to define cell size mutants).
(0.06 MB DOC)

List Data S1 GeneList file of the GenePix ArrayList v1.0 format describes the microarray features of Figure 1A. The sequence of the barcode probe is used as the unique “ID” and the yeast gene name is used as the “Name” of the probe. The letter ‘X’ at the 5′ of the probe sequence denotes the C3 or C6 amino-linker; the ‘_UP’ suffix attached to the yeast gene name indicates the ‘UP’ barcode tag, where the absence of suffix indicates the ‘DN’ tag.
(0.07 MB TXT)

Data S1 All on-chip replicate averaged Z scores for Agilent and barcode experiments.
(1.26 MB XLS)

Data S2 Numerical data used to generate Figure 3E. The yeast cell size cluster was derived from reference [28].
(6.45 MB ZIP)
