# Supplementary material for: Systematic Validation and Atomic Force Microscopy of Non-Covalent Short Oligonucleotide Barcode Microarrays
Source: PLoS One. 2008 Feb 6;3(2):e1546. doi: 10.1371/journal.pone.0001546 (PMC2212718; doi:10.1371/journal.pone.0001546)
Supplement: Supplemental Text S1 — Detailed description of a) supplementary scatter plots of SUBarray and Agilent replicate arrays, and the methods for analysis of b) barcode microarray data and c) atomic force microscopy data are included in this supplemental text. (0.06 MB DOC) [file pone.0001546.s001.doc]

**Supplementary materials**

**Supplementary scatter plots of SUBarray and Agilent replicate arrays.**

While the deviation between Agilent replicates is much lower (compare Figures S4, S5), we observed a large number of anti-correlated dye-swap artifacts with our Agilent microarray experiments (Figure S5A) not present in the SUBarray comparisons. These artifacts occurred in a common set of barcodes in each of two independent experiments using different elutriated population DNA samples. There was no obvious sequence enrichment in this population.

A common source of artifacts in barcode microarrays, largely due to the small size and efficiency of amplification of the barcode amplicons, is PCR contamination. However, these dye-swap artifacts occurred in both UP and DN reactions equally, and PCR contamination would not be expected to produce anti-correlated spots. Further, these anti-correlated spots were not a product of poor filtering, as they ranged from low to very high in signal intensity. While it is possible that the published conditions for another barcode platform that we employed (custom produced with an inkjet synthesizer, [2]) were not correct for these Agilent arrays, there was nonetheless high correlation between intra-experimental replicates in which a common dye-labeling scheme was used (Figure S5B) as well as between appropriately correlated spots in dye-swap experiments (Figure S5A). We reason, therefore, that these defects are not due to poor hybridization conditions, in general.

**Microarray analysis:**

**Data normalization:**

Raw microarray data was visually inspected to remove obvious signal artifacts. Since UP and DN barcode tags are amplified in separate PCR reactions, they were separated and treated independently. Local background subtracted signal intensities were used to compute average log2 intensities (A = [log2(Cy5)+log2(Cy3)]/2) and log2 enrichment ratios (M = log2[Cy5/Cy3]), which were then normalized by block using the LOWESS algorithm within the program Vector Xpression 3 (Invitrogen/Informax). Background subtracted intensities with values less than zero were assigned an arbitrary value of 1 (log2 = 0).

**Filtering by intensity:**

***Methodology:***

Rather than applying a constant intensity threshold for significant calls, we chose to use an optimization strategy to guide two-step filtering thresholds, first filtering on an individual array basis, and second across multiple barcode tags and arrays. We chose thresholds to maximize properties associated with high quality microarray data, including high recovery of true positive data, low false positives, low replicate standard deviation and Pearson's correlation coefficient between technical experimental replicate arrays.

Using a progressively increasing signal to noise ratio (SNR) threshold, we generated a Receiver Operating Characteristic (ROC) curve to show the rates of loss of both true and negative data (Figure S2A). The point of maximum loss of false positive (%FP) data with maximum retention of true positive data (%TP), occurs when the rates of loss of true positives and false positives are equal:

d(%TP)/dx = d(%FP)/dx where x is the thresholding criterion employed

defined by the function:

df(x)/dx = d(%TP)/dx - d(%FP)/dx = 0

The point where these two rates are equal, and df(x)/dx = 0, occurs at the maximum of the function:

f(x) = %TP - %FP

This is also the 45˚ tangent to the ROC curve shown in Figure S2A.

Similarly, employing the Pearson's correlation coefficient (r) and the standard deviation between corresponding spots on replicate arrays (SD_average_) in the equation f(x) in place of %FP yielded similar thresholds. In these cases, the optimum threshold is represented by the point at which the function f(x) begins to plateau (Figure S2B):

f(x) = %TP - SD_average_/SD_average,initial_

f(x) = %TP - (1-r)/(1-r_init_)

In our analyses, we chose to apply equal weighting to %TP and %FP, but this could be varied:

f(x) = c[%TP] - (1-c)[%FP] where 0 ≤ c ≤ 1

***Defining false positives and false negatives:***

Our experiments utilized the haploid yeast deletion collection, which is comprised of all viable non-essential, but not essential deletion strains. As such, there are a large number of absent strains and barcodes available as negative controls on the microarray. We defined all non-essential barcodes representing strains present in the experimental pool as true, and all those essential barcodes absent from the pool as false. Because insignificant hybridization signals might result due to loss of barcode targets during amplification from the pool, mutations in the barcode cassettes, or other unknown reasons, the sensitivity of our microarrays is an underestimate, and the number of true barcodes is lower than defined.

***Criteria for measuring significant signal:***

We compared absolute intensity, background subtracted intensity, and signal-to-noise ratio (SNR) in filtering our microarray data. For our arrays, background subtracted intensity and SNR performed equally well, while absolute intensity was much worse at distinguishing false positives and true positives (not shown). Due to variability in our background signals, we chose to use SNR thresholds. For Agilent arrays, which are constructed on a surface that allows little background target binding and thus low background variation, background subtracted intensity thresholds performed best and were used in our analyses (not shown).

***Defining individual array specific intensity thresholds (step 1):***

We defined thresholds as fractions of the mean SNR (or background subtracted intensity) for UP or DN tag spots across the array in each channel. The number of true and false barcode spots above each sequential increasing threshold in at least one channel were used to define %TP and %FP, and define the function f(SNR)=%TP - %FP. The SNR threshold corresponding to a maximum in the function f(SNR) was chosen for the specific array. This process was repeated for all microarrays.

For these Agilent arrays, which contain only haploid yeast deletion barcodes, essentially all of which were present in our pools, we could not use false positives as a measure of appropriate filtering thresholds. As such, we used a measure of the Pearson's correlation coefficient (1-r) between replicate dye swap experiments, relative to the initial unfiltered correlation coefficient (1-r)_initial_:

f(intensity) = %TP - (1-r)/(1-r)_initial_

The optimal thresholds were similar when the average inter-replicate standard deviation was used (SD_average_/SD_average,initial_). In the case of our arrays, these measures yield similar thresholds to %FP (Figure S2B).

***Defining experimental pool specific intensity thresholds (step 2):***

Significant near noise signal intensities resulting from random hybridization, as in the case of false positive signals, should occur randomly across multiple replicates and multiple arrays. In contrast, those due to *bone fide* true positive hybridization should occur more consistently.

We found that the total number of occurrences of significant barcode replicate spots (#SRs; as defined by array specific thresholds) across all arrays, for both UP and DN barcodes, was best at distinguishing true positive from false positive gene deletion strains (typically 2 replicates per each of 2 barcodes, per 6 arrays, for a total of 24 possible significant signals). This is as compared to using the fraction of total possible barcodes detected (%SRs), or the best of the two UP or DN barcodes alone (Figure S2D). It must be noted that the relative performance of these criteria (#SRs versus %SRs) varies with the number of experiments performed, and should be examined prior to application.

Similar to our array specific thresholds, we defined our experimental pool specific thresholds by maximizing the function:

f(#SRs) = %TP - %FP

Since we could not apply this method to these Agilent arrays, we applied a filter of at least 2 (of 4) significant occurrences for any given barcode replicate spot.

This filtered data was used for all further analyses.

**Filtering by relative enrichment:**

***Converting data to Z scores:***

To compare data from multiple array experiments, we converted log2(background subtracted median Cy5/Cy3) data into Z scores. UP and DN tags barcode Z scores were calculated separately and combined (Z_UP_ = [log2_replicate_-average log2_UParray_]/SD_log2,UParray_). Data from all experiments consistently reflects the relative enrichment of the elutriated fractions (elutriated/initial population; Z score > 0, enriched; Z score < 0, depleted).

***Defining false positives and false negatives:***

We defined our thresholds for enrichment similarly to our intensity thresholds, applying an analogous two step filter. To define significantly enriched or depleted barcodes, we first defined high confidence cell size false and true gene deletion strains and barcodes using data obtained from systematic cell size measurements with a Coulter Z2 particle analyzer. False gene deletion, or wild-type strains, were defined as those that confer a cell size deviating by less than 0.2 SDs from the average mean, median, and modal cell size of the haploid deletion set. True gene deletions were defined as those that conferred a cell size at least 1 SD larger (*lge*) or smaller (*whi*) than the average mean and median cell size.

***Defining individual array specific Z score thresholds (step 1):***

In our definitions, positive and negative Z scores correspond to enrichment (*whi* strains). and depletion by elutriation (*lge* strains), respectively. Because the strength of the selection (elutriation flow rate) independently determines the degree of enrichment or depletion, we set positive and negative Z score thresholds separately.

We used the number of false and true barcode spots above sequentially higher Z score thresholds to define %TP, %FP, and the function f(Z score) = %TP - %FP. This function, as well as an ROC plot, was used to evaluate the effectiveness of different methods of filtering (Figure S3A). However, we chose to use only the wild-type (or false) gene deletion subset in defining our thresholds. Since the systematic and barcode experiments rely on different criteria to identify cell size defects (median/mean versus small G1 cells), a strong phenotype in one experiment is not necessarily strong in the other. However, wild-type distributions do not vary in either criteria and should be constant between the experiments. We chose positive and negative Z score thresholds that included less than 2.5% (5% total) of significant wild-type barcode detection. Notably, these values were similar to the 45˚ tangent to the ROC curves.

***Defining experiment specific enrichment thresholds (step 2):***

Replicate averaging is widely known to decrease data variability, but it nonetheless results in loss of information. Moreover, it is complicated by the occurrence of outliers, which either necessitate elimination of data, or inclusion of skewed data. We defined thresholds based on the total number of significant replicates observed (#SRs; as defined by array specific thresholds) for both UP and DN tags of a given deletion strain. Positive and negative Z scores were treated separately. We found that using the #SRs performed better than other measures (Figure S3A). Similar to our step 1 filter, we defined thresholds for positive or negative Z score data that included less than 2.5% (5% total) of wild-type gene deletion strains.

As one final filtering step, to partially account for any dye-swap artifacts, we determined the number of *lge* or *whi* strains identified incorrectly by the reciprocal Z score as *whi* or *lge*, respectively, and increased the threshold where necessary to reduce these false identifications to less than 2.5% (5% total).

Note. The algorithms are summarized in the accompanying flow charts.

**Atomic force microscopy analysis:**

**Criterion in choosing scan regions:**

From our fluorescent scans of the microarray surface, the highest target fluor concentration was observed at the edges of the spotted and hybridized probe, due to the "coffee stain effect" caused by capillary flow upon drying [3]. As such, our scans were focused in on the edges, and the scanned regions were chosen to have a similar density of probe across all conditions.

**Conversion of data to absolute height:**

Voltage measurements within the 1 micron x 1 micron scanned regions, divided into 512x512 pixels, were converted to height [with the zero level height positioned to the average height across the region] using the formula:

Height = Voltage * Z scale * Z scan sensitivity / 2^8*Bytes/Pixel^

**Reduction of background effects:**

***Estimation of the non-background pixel distribution:***

From an examination of a region of glass surface lacking bound probe, we observed a largely symmetrical distribution of pixel heights reflecting the background, spanning approximately 1 nm across (Figure S10A). By subtracting the function created by reflecting the left half of the pixel distribution across its modal point, we generate a residual of "non-background" pixels (Figure S10B). As compared to the no probe condition, the probe containing regions display a clear distribution of pixels, representing the residual difference from the distribution of the background. Also, there is an increased frequency of larger pixel heights in the complementary plus target sequence condition, as observed in the background corrected data.

***Reduction of surface height variation effects:***

Given the large variation in height attributed to background alone, we applied an algorithm implemented in PERL to reduce this variation. We divided the 512 x 512 matrices into 512 vertical or horizontal slices. As part of the algorithm, we identified the local minima in each slice. The minima were identified by a positive concavity, a change from negative to positive slope spanning the point, and a minimum value relative to the adjacent two pixels on either side. Minima were used to set a new background (Figure S10C). The average background calculated using either vertical or horizontal slices was used to define the new background and to calculate the new peak heights. Comparison of a cumulative histogram of pixel height for the no probe condition shows that the average background variation is reduced by approximately half, but is not eliminated by this method (Figure S10D).

**Calculation of peak characteristics:**

***Choice of a cut-off for peak identification:***

We chose to use 0.6 nm as a peak height cut-off as this point eliminates 99% of pixels in the no probe condition. Any pixels below this height were ignored in calculations of peak characteristics.

***Definition of peak boundaries:***

Peaks were defined using maxima in the matrices with minimum heights of 0.6 nm. The dimensions of each peak were defined by counting the number of pixels above 0.6 nm, up to 6, in each direction (Left, right, up, down) from the defined peak maximum. The number counted in each direction represents the dimensions of the rectangular window defining each peak. The total pixels above 0.6 nm within the boundaries of this window were then counted to define the peak dimensions.

***Calculation of peak diameters and average peak heights:***

The total number of pixels within each peak window was used to calculate the 2-dimensional area of each peak using the formula:

Area = # pixels * area/pixel = # pixels * (1000nm / 512 pixels)^2^

The area was then converted into a diameter by estimating each peak base as a circle:

Area = πr^2^

Peak diameter = 2r = 2 * (Area / π)^1/2^

The average peak height was calculated by dividing the total height of all the pixels above 0.6 nm in each peak window by the total number of pixels.

Average peak height = ∑pixel heights / #pixels

***Calculation of peak statistics and bound / unbound cut-offs:***

The measures calculated as described above were used to define mean and median peak attributes. 95% and 99% cut-offs were defined using cumulative histograms of individual peak attributes (Figure 5E, F of the main body).

**Supplementary reference**

1. Eason RG, Pourmand N, Tongprasit W, Herman ZS, Anthony K, et al. (2004) Characterization of synthetic DNA bar codes in Saccharomyces cerevisiae gene-deletion strains. Proc Natl Acad Sci U S A 101: 11046-11051.

2. Ooi SL, Shoemaker DD, Boeke JD (2001) A DNA microarray-based genetic screen for nonhomologous end-joining mutants in Saccharomyces cerevisiae. Science 294: 2552-2556.

3. Deegan RD, Bakajin O, Dupont TF, Huber G, Nagel SR, et al. (2000) Contact line deposits in an evaporating drop. Phys Rev E Stat Phys Plasmas Fluids Relat Interdiscip Topics 62: 756-765.
