# Supplementary figures and images for: Systematic Validation and Atomic Force Microscopy of Non-Covalent Short Oligonucleotide Barcode Microarrays
Source: PLoS One. 2008 Feb 6;3(2):e1546. doi: 10.1371/journal.pone.0001546 (PMC2212718; doi:10.1371/journal.pone.0001546)

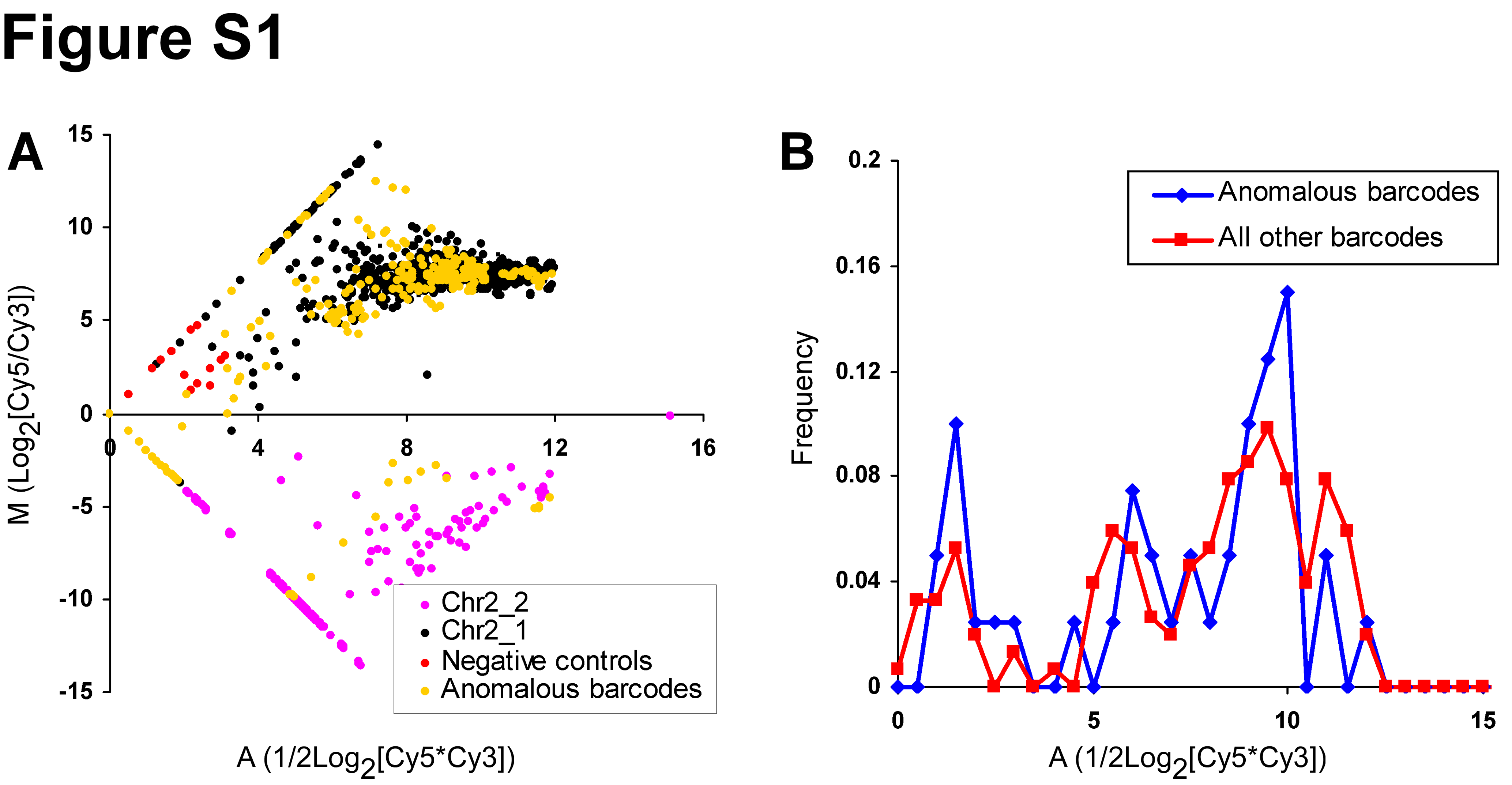

Supplement: Figure S1 — Analysis of previously defined anomalous barcodes [1]. A. The log2 ratio of the Cy5/Cy3 (M) channels is plotted versus the average log2 value of the signal intensity in each channel (A) for 8 replicates of each barcode. Barcodes from sub-populations of non-essential deletion strains from chromosome 2 are labeled with Cy5 (Chr2_1; black) and Cy3 (Chr2_2; magenta). Negative control sequences are shown (red). Previously defined anomalous barcodes are plotted in gold. B. Frequency of the average log2 value of the signal intensity from each channel for anomalous and all other barcodes. Barcode values are an average of all replicates. (0.89 MB TIF) [file pone.0001546.s002.tif]

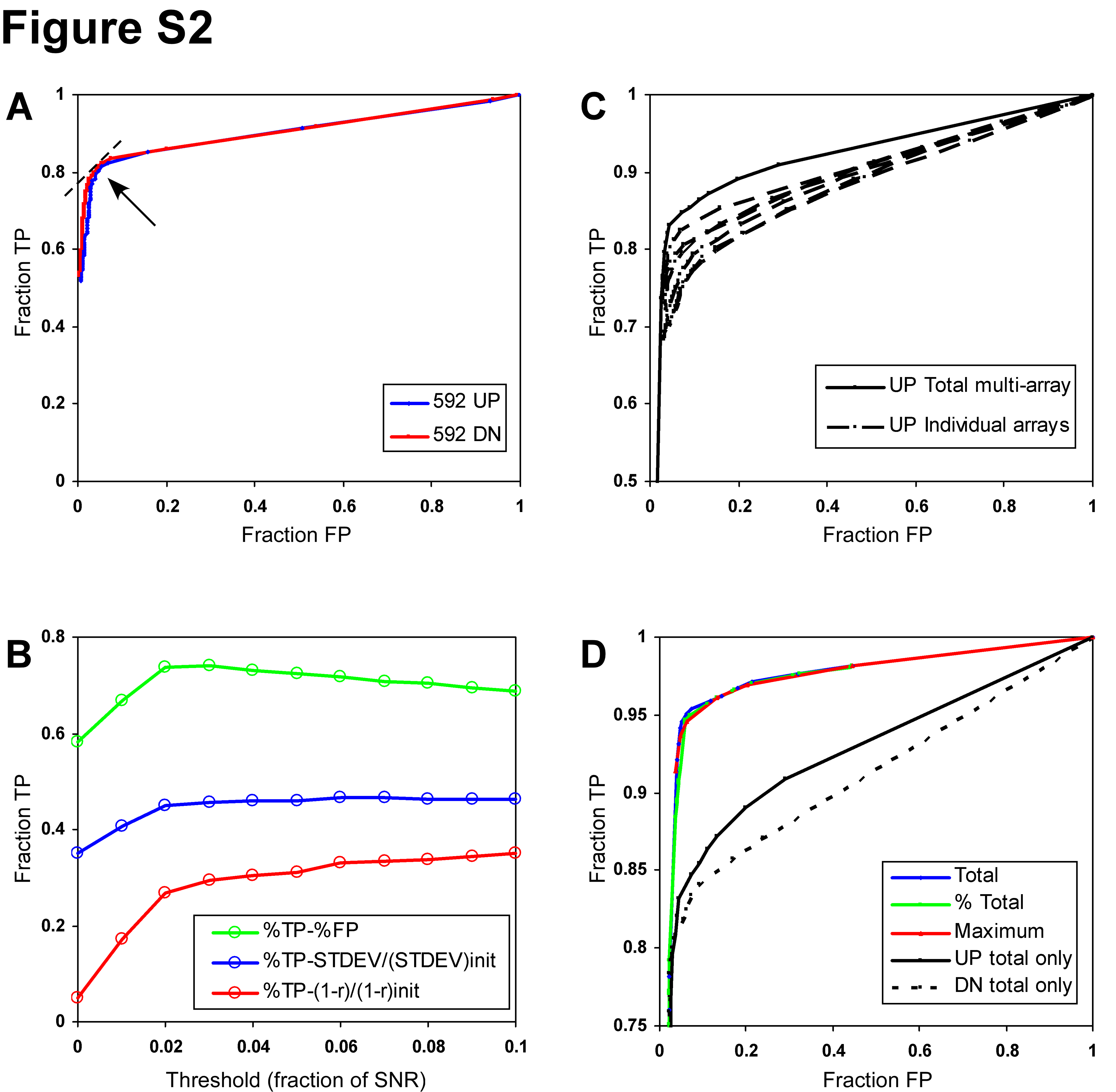

Supplement: Figure S2 — Use of ROC curves in defining intensity thresholds. A. True and false barcodes or genes are defined based on their presence or absence from the experimental pools, respectively. The 45{degree sign} tangent (dashed black) to the characteristic ROC plots (red, blue) is the point at which the rate of loss of false positives equals the rate of loss of true positives. B. Comparison of SNR thresholds defined by different measures of microarray data quality. Dotted line indicates the maximum obtained using true positive and false positive data (green) to set thresholds. Plots obtained from the measures of the average standard deviation (STDEV) between analogous spots on different arrays (blue) or the Pearson's correlation coefficient between arrays (red) begin to plateau at a similar threshold. A representative comparison between two dye-swap replicates is shown for all methods. C. Filtering according to the total number of barcodes with significant signal across multiple arrays (solid), rather than by the SNR from individual arrays (dashed) increases the ability to distinguish false and true data. Data is plotted for UP tags, but DN tag ROC plots are analogous. D. Filtering according to the total number of barcodes with significant data (Total; blue) yields slightly better data than using the fraction of total barcodes (% Total; green) or the maximum number of significant replicates for the best of the UP or DN barcode only (Max; red), or either the UP (black) or DN (black dashed line) tag data alone. (1.49 MB TIF) [file pone.0001546.s003.tif]

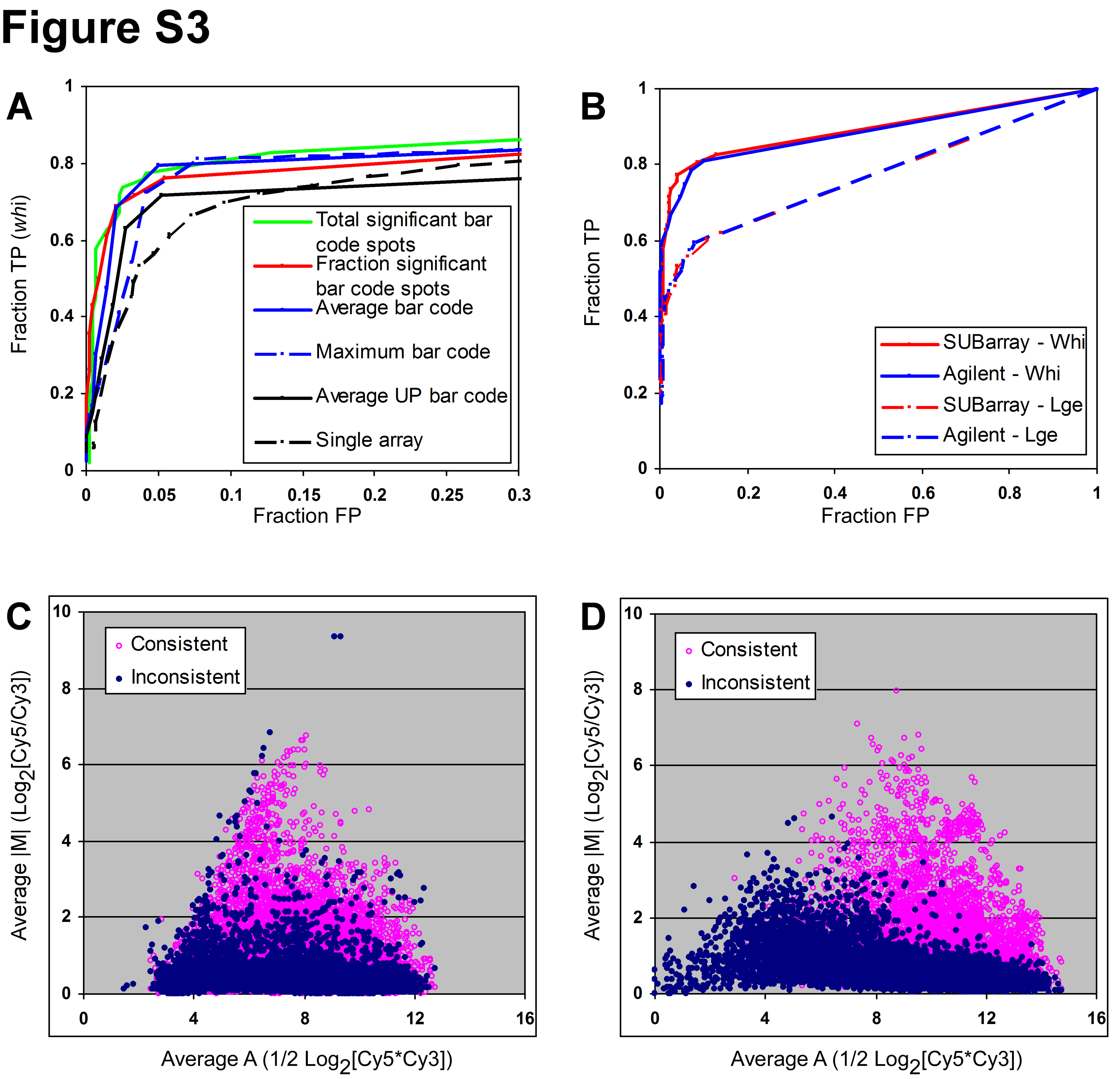

Supplement: Figure S3 — Use of ROC plots in defining Z score thresholds for significant hits. A. True positive (either whi or lge) and false positive (wild-type) genes are defined based on systematically confirmed size characteristics. Individual array Z score thresholds (dashed black) are defined at the point that removes at least 95% of the false positive genes. Filtering by the total number of significant values across all arrays for both barcodes (green) yields better data than the fraction of possible hits (red), or the average (blue) or maximum (dashed black line) Z score for both UP (black) or DN (not shown) barcodes. ROC plots are shown for whi strains only. Data are analogous for lge strains. B. Comparison of the performance of optimized filtered data from Agilent (blue) or SUBarrays (red) for both whi (solid) and lge (dashed) strains. C. Dye-swap analysis from Agilent (left) and SUBarrays (right). Dye swap and technical replicate spots with consistent enrichment or depletion by elutriation (magenta) or those with any inconsistent values (blue) are plotted on a graph of the average absolute value of the log2 ratios (M) versus the average log2 value of the signal intensities (A). Barcodes with high intensities and high log ratios are the most consistent. Inconsistent high intensity, high log ratio barcode replicates represent dye swap artifacts and are more frequent in these Agilent arrays. Comparisons of Agilent and SUBarrays are between two and four replicate experiments, respectively. Consistent values agree for all experiments. (2.60 MB TIF) [file pone.0001546.s004.tif]

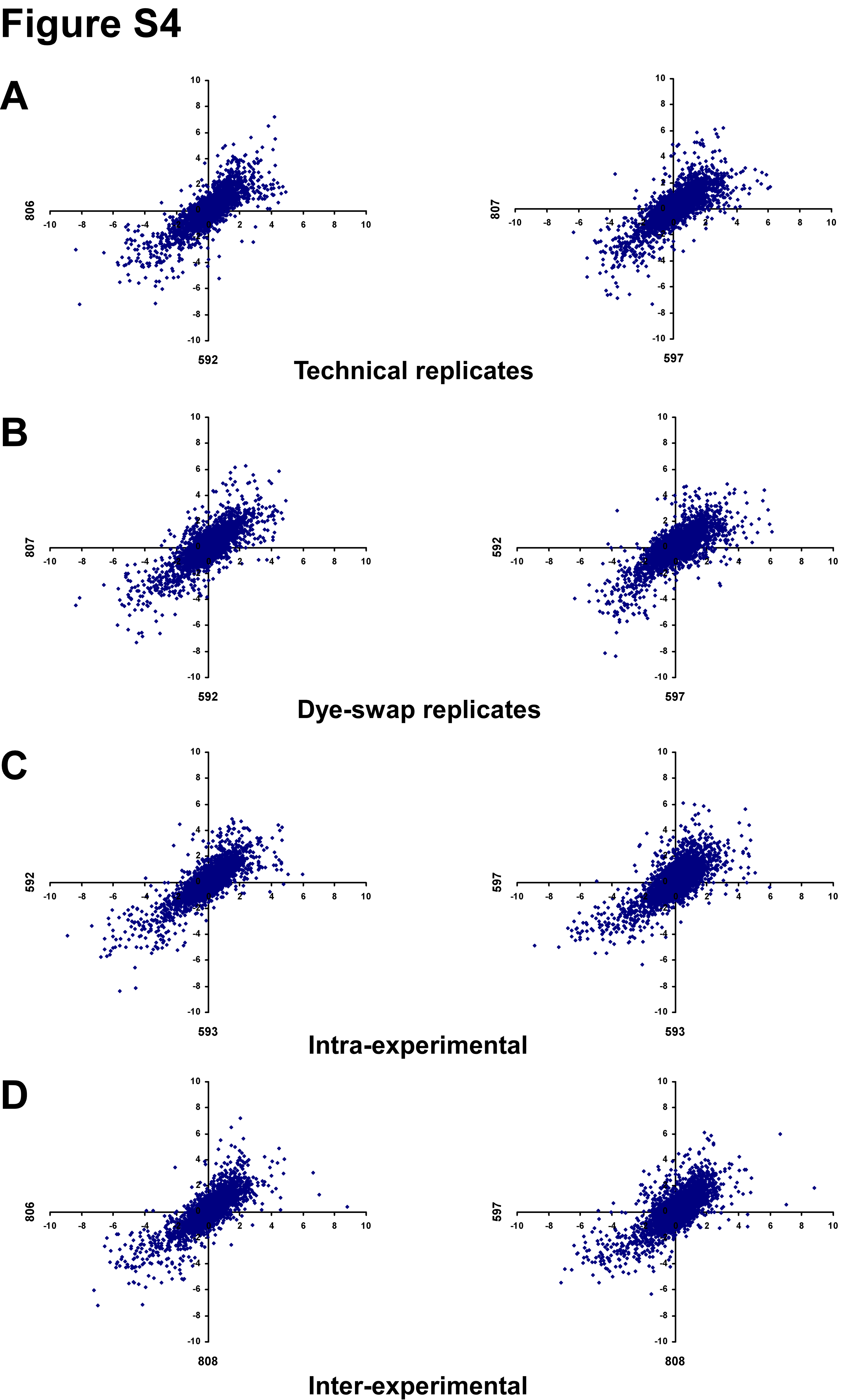

Supplement: Figure S4 — (A–D) Scatter plots for the best (left) and worst (right) correlated SUBarray replicate arrays (as defined by Pearson's correlation coefficients) for technical (A), dye-swap (B), and intra- (C) or inter-experimental (D) replicate arrays. Z scores (as defined in the text) from on-chip replicate spots were averaged prior to generation of the scatter plot. Each experiment represents an independent set of PCR reactions. Dye-swap Z score values were multiplied by a factor of -1. All array data are derived from a log2 ratio of elutriated/pre-elutriated samples. Arrays are defined as: 592 (E2-21ml/min, log2[Cy5/Cy3]); 593 (E2-24ml/min, log2[Cy5/Cy3]); 597 (E2-21ml/min, log2[Cy3/Cy5]); 806 (E2-21ml/min, log2[Cy5/Cy3]); 807 (E2-21ml/min, log2[Cy3/Cy5]); 808 (E1-24ml/min, log2[Cy5/Cy3]). Dye-swap replicates are italicized. (2.03 MB TIF) [file pone.0001546.s005.tif]

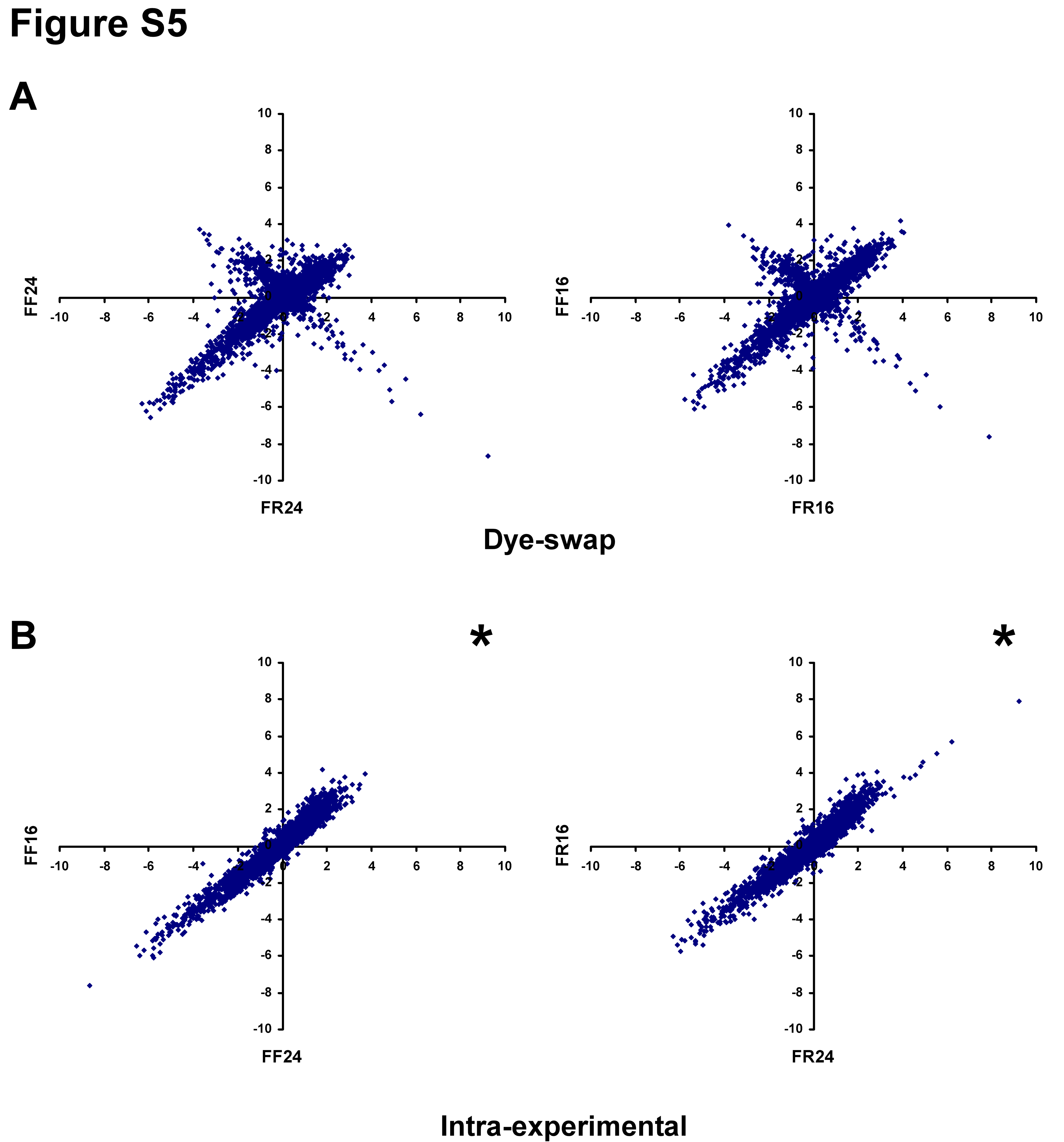

Supplement: Figure S5 — (A–B) Scatter plots for the best (left) and worst (right) correlated Agilent replicate arrays (as defined by Pearson's correlation coefficients) for dye-swap (A) and intra-experimental (B) replicate arrays. An intra-experimental comparison between two arrays with the same labeling scheme is indicated by an asterisk and represents an estimate of technical replication; the average Pearson's correlation coefficient is listed in Table 1 (Average r = 0.95). Comparisons were executed as in Figure S4. Arrays are defined as: FR16 (E3-16ml/min, log2[Cy5/Cy3]); FF16 (E3-16ml/min, log2[Cy3/Cy5]); FR24 (E3-24ml/min, log2[Cy5/Cy3]); FF24 (E3-24ml/min, log2[Cy3/Cy5]). Dye-swap replicates are italicized. (1.38 MB TIF) [file pone.0001546.s006.tif]

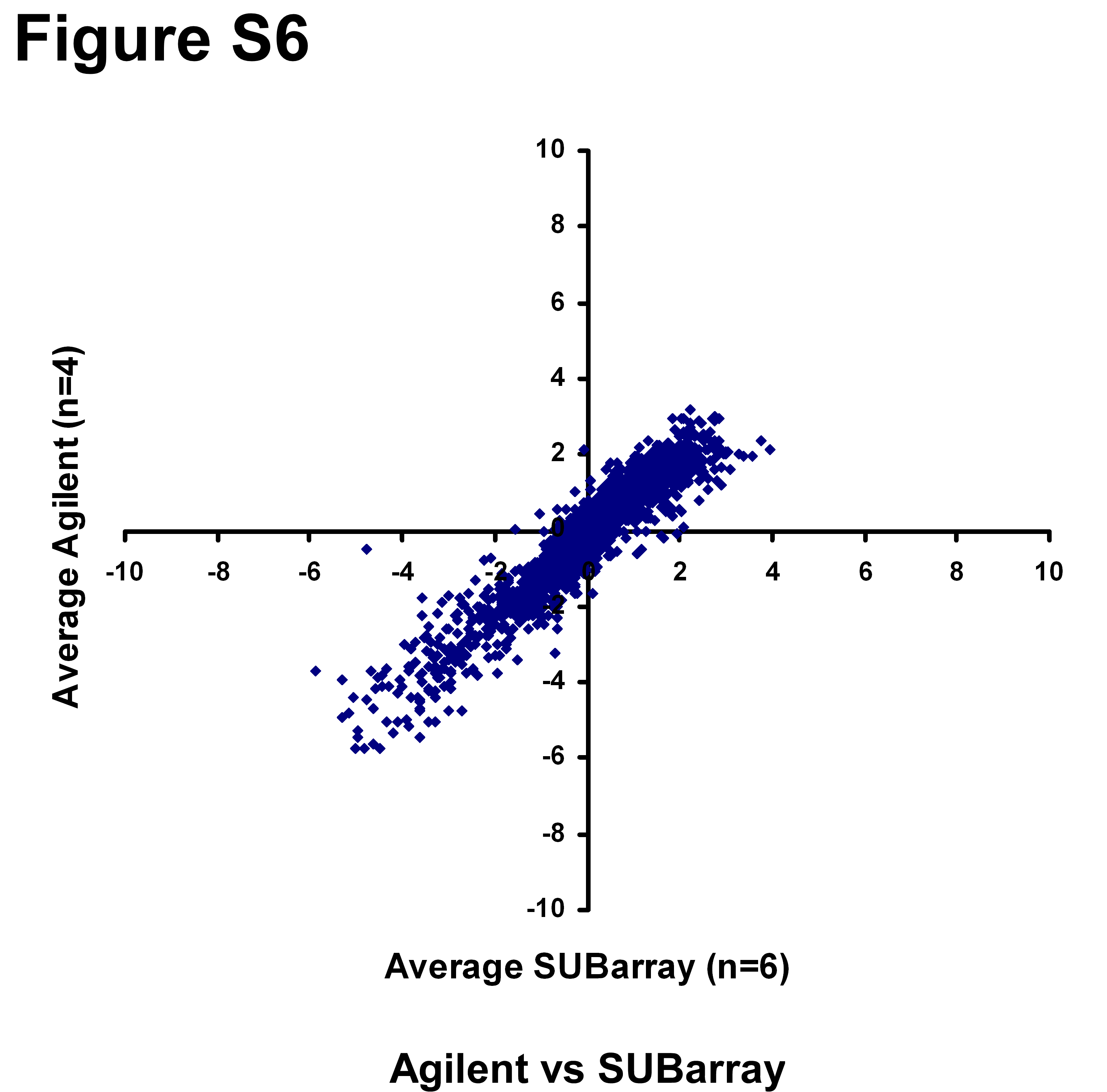

Supplement: Figure S6 — Scatter plot of the average Z scores from Agilent (y-axis) and SUBarrays (x-axis). Z scores were averaged from all arrays (Agilent, n = 4; SUBarray, n = 6). Comparisons were executed as in Figure S4. (0.12 MB TIF) [file pone.0001546.s007.tif]

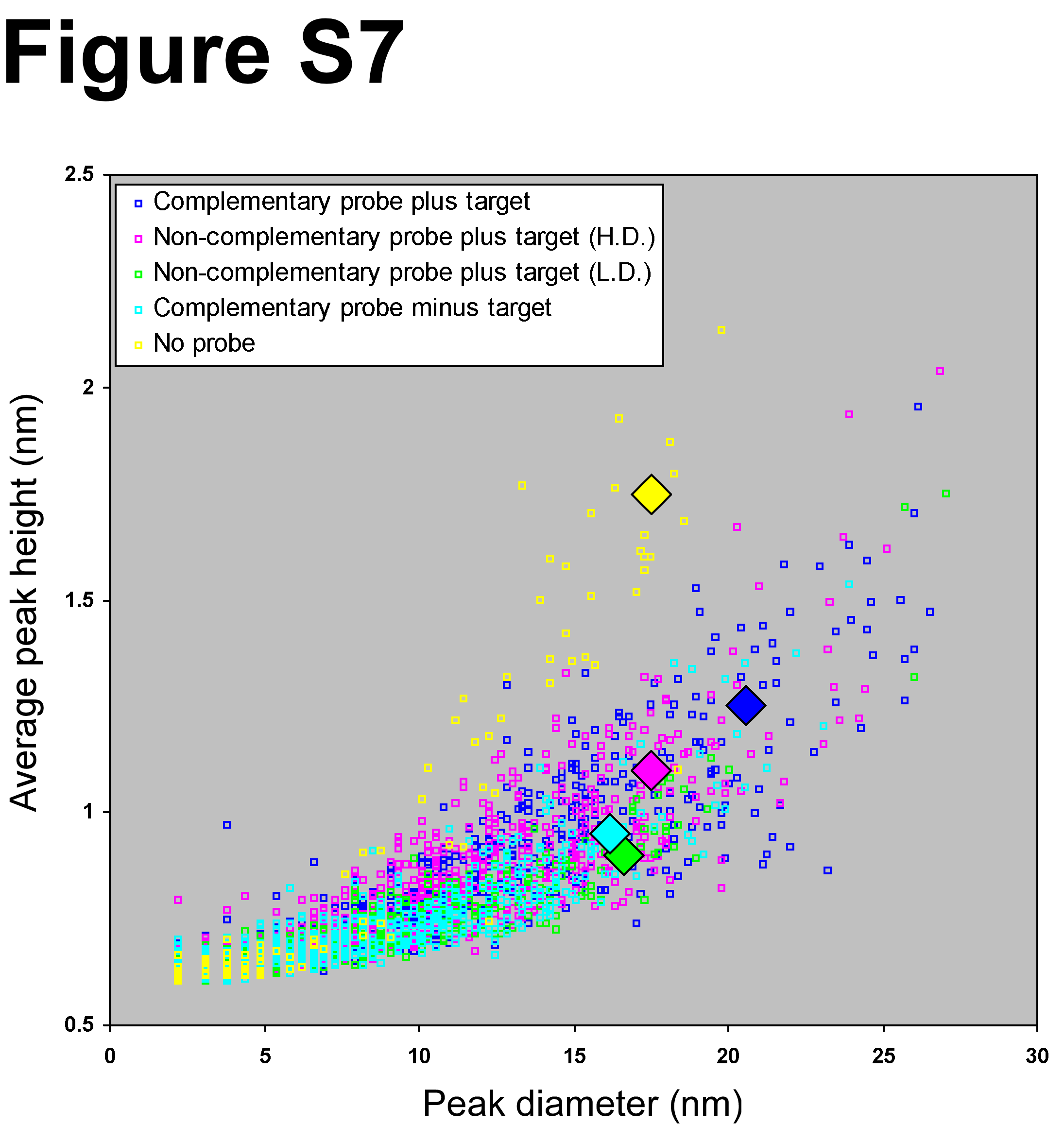

Supplement: Figure S7 — Scatter plot of the dimensions of all peaks (squares) identified by atomic force microscopy. 95% exclusion limits for each scan area are shown as colored diamonds. Peaks from a region lacking any probe (yellow) are less numerous (n = 103) and have significantly different properties than all other peaks. (0.16 MB TIF) [file pone.0001546.s008.tif]

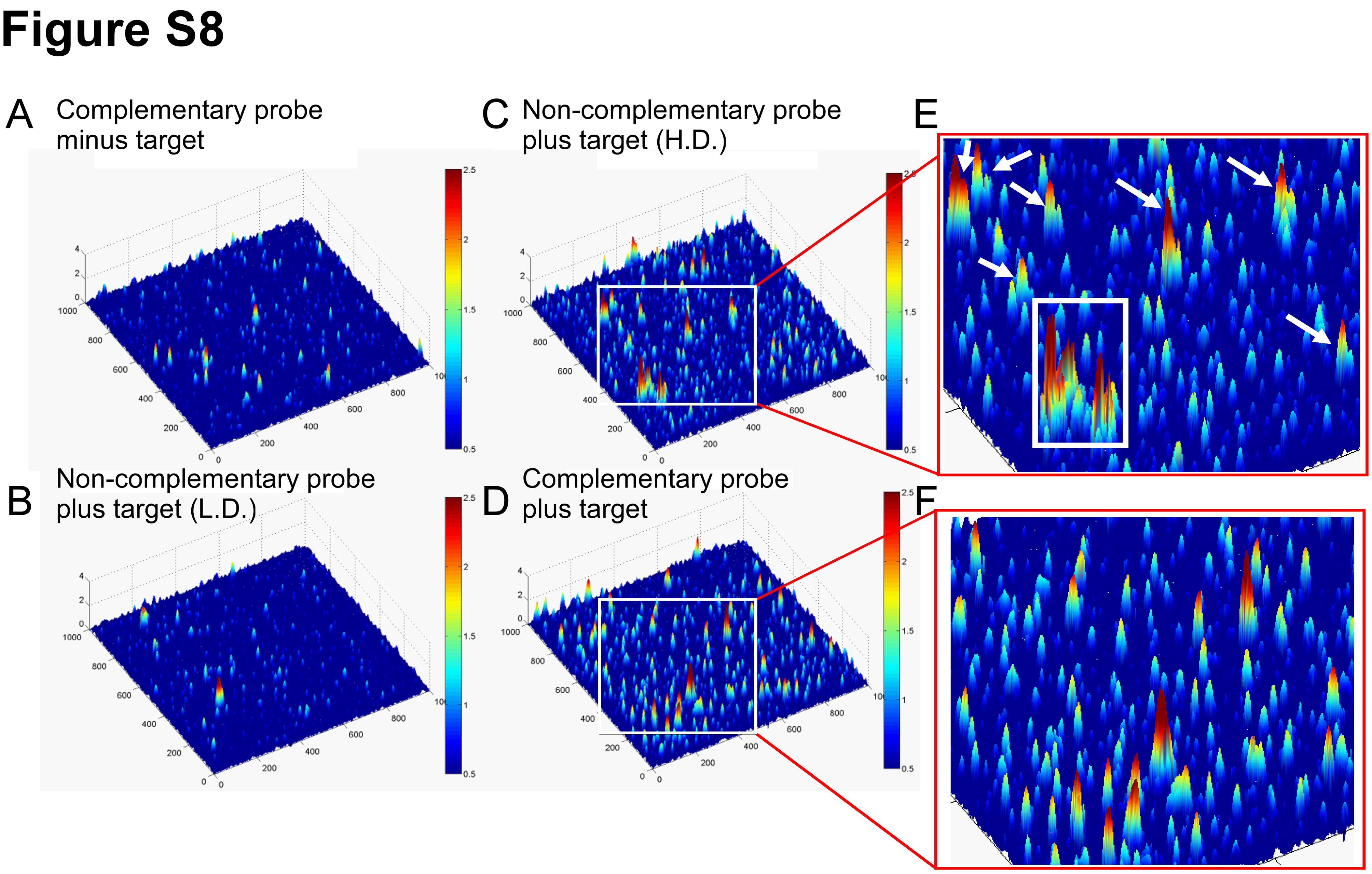

Supplement: Figure S8 — Background adjusted plots of the AFM scan regions. Data from original scans was adjusted to reduce background variation in the height of the glass surface. Peak height for each region is color coded to height. Both complementary probe minus target (A) and low density non-complementary probe plus target (B) show few large height peaks, while high density non-complementary probe plus target (C) and complementary probe plus target (D) show many more. However, the majority of the large height peaks in the high density non-complementary condition appear to have shoulders (E, white arrows), while the those in the complementary condition do not (F), suggesting that these peaks may be an artifact of high density, and represent two juxtaposed peaks. The region boxed in E has very different surface arrangement than any other peaks observed, is marked in Figure 4C as aberrant, and was omitted in all analyses. (7.58 MB TIF) [file pone.0001546.s009.tif]

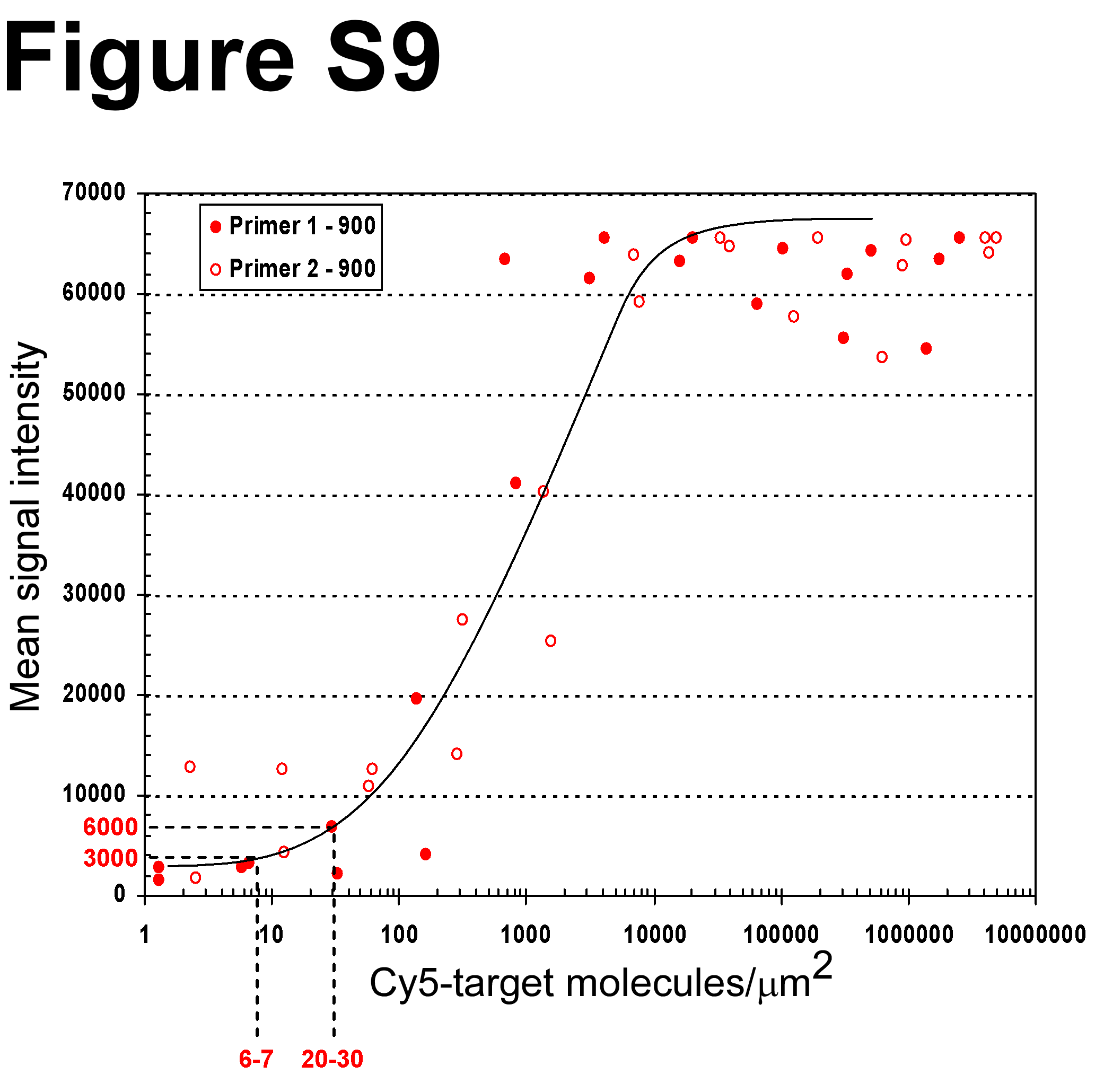

Supplement: Figure S9 — Fluorescence calibration curve for Cy5-target abundance. Replicate slides with known dilution series of one of two Cy5-labelled UP primers were scanned under comparable PMT and power settings to the AFM run. Mean signal intensities for each spot were plotted versus the total number of target molecules per total spot area (μm2). The potential range of signal intensities (3000–6000) for the scanned AFM region was used to estimate the theoretical target concentration (6-30; equivalent to hybridized probe-target peak number) in the scanned AFM region. (0.10 MB TIF) [file pone.0001546.s010.tif]

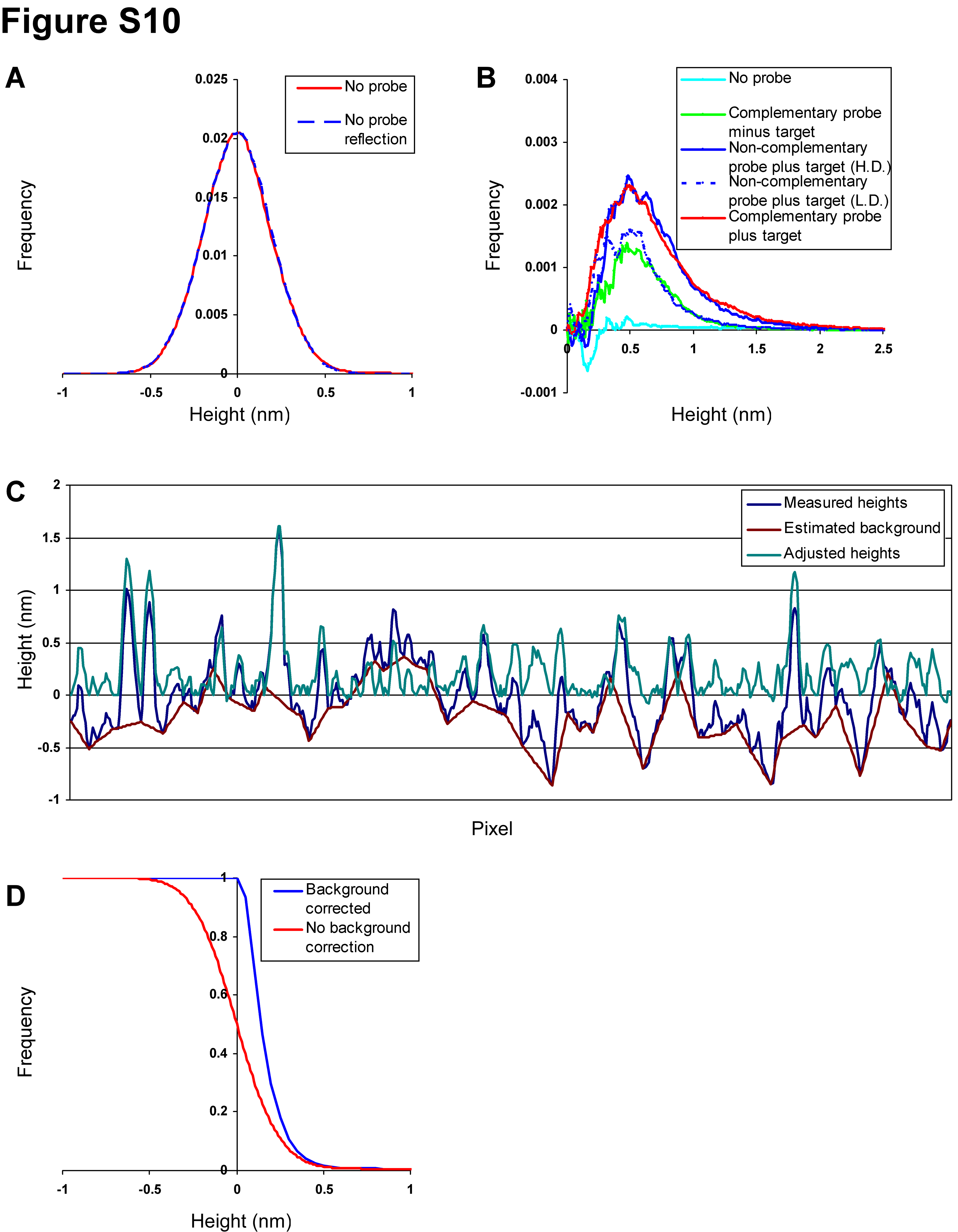

Supplement: Figure S10 — Pixel and adjusted analysis of AFM data. A. Pixel distribution for a scanned region containing no probe (red). Shown for comparison is the left side of this distribution reflected across its modal point (blue dashed) B. Residual distribution of scan regions after removal of reflected background distributions. To remove an aberrant region containing abnormal peaks, histogram represents only half of the high density non-complementary probe containing region. C. Vertical strip of AFM scan before (blue) and after (green) removal of estimated background (brown). D. Pixel distribution before (red) and after (blue) background correction. (1.53 MB TIF) [file pone.0001546.s011.tif]

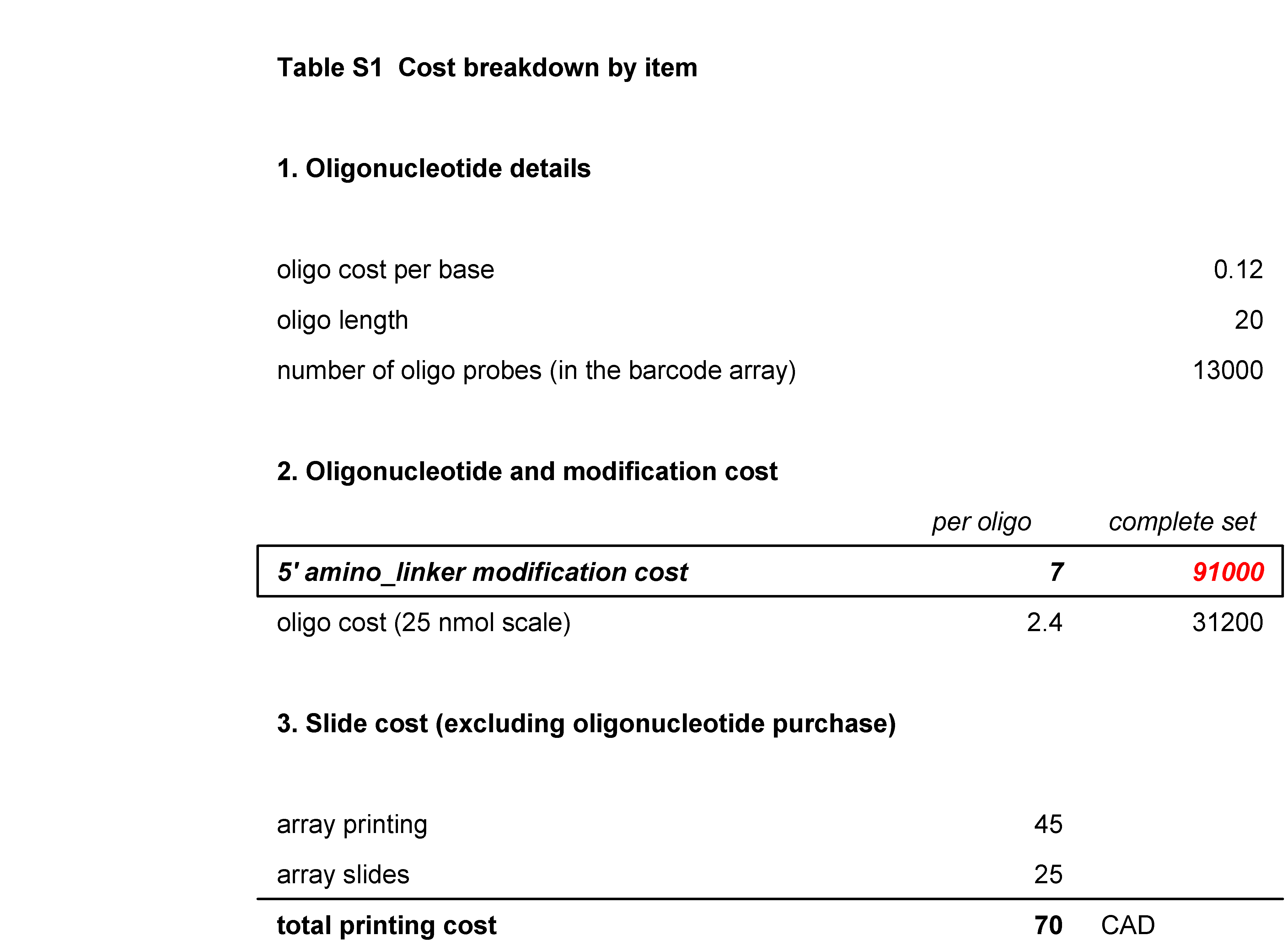

Supplement: Table S1 — Breakdown of microarray construction cost by item. (0.12 MB TIF) [file pone.0001546.s012.tif]
