# Supplementary material for: Systematic Validation and Atomic Force Microscopy of Non-Covalent Short Oligonucleotide Barcode Microarrays
Source: PLoS One. 2008 Feb 6;3(2):e1546. doi: 10.1371/journal.pone.0001546 (PMC2212718; doi:10.1371/journal.pone.0001546)
Supplement: Supplementary file 3 [file pone.0001546.s013.doc]

Intensity filter (to generate present and absent calls)

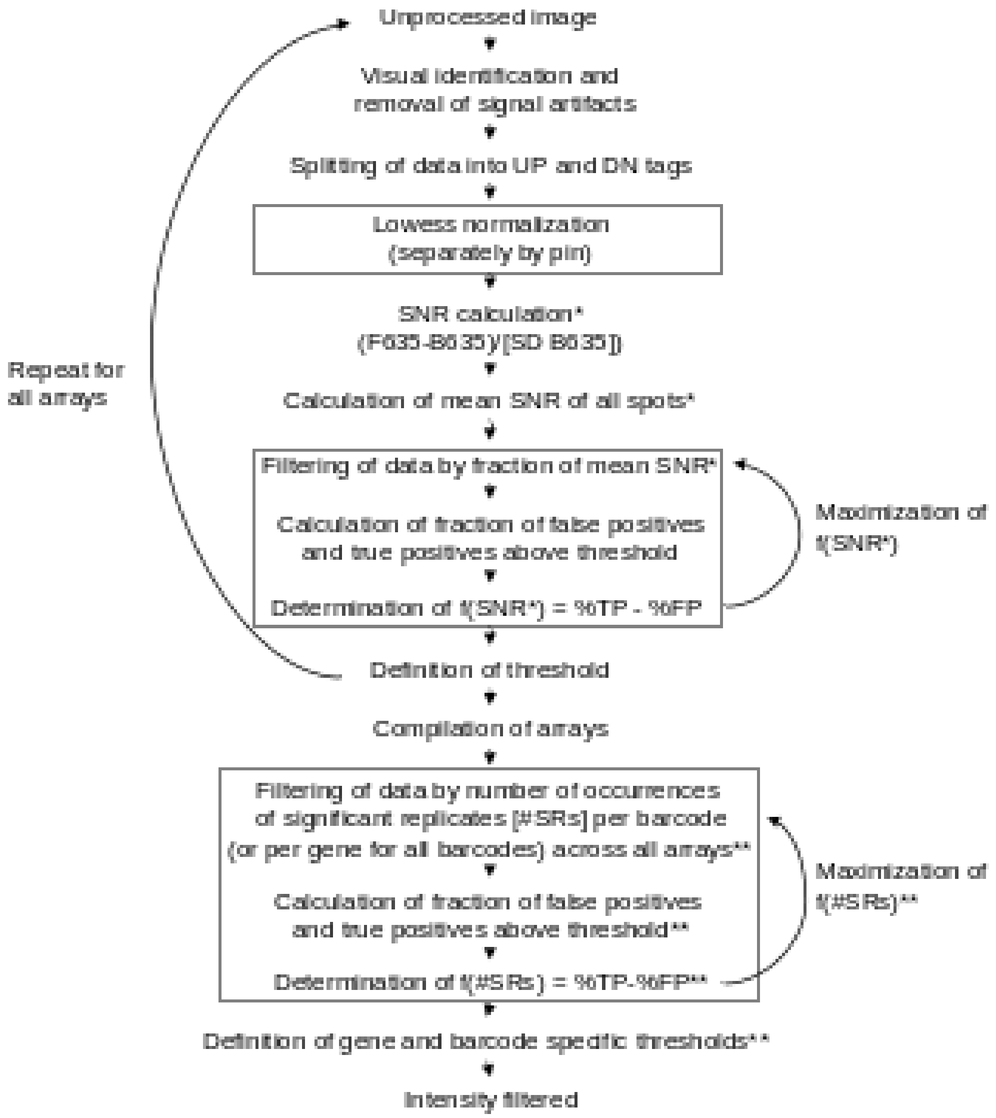

*For Agilent arrays, we filtered based on background subtracted median intensity, as this proved to be the optimal measure for these arrays

**For Agilent arrays, lacking appropriate criteria to optimize, we filtered out features with less than two representations across 4 replicate microarray experiments.

Z score filter (to define cell size mutants)

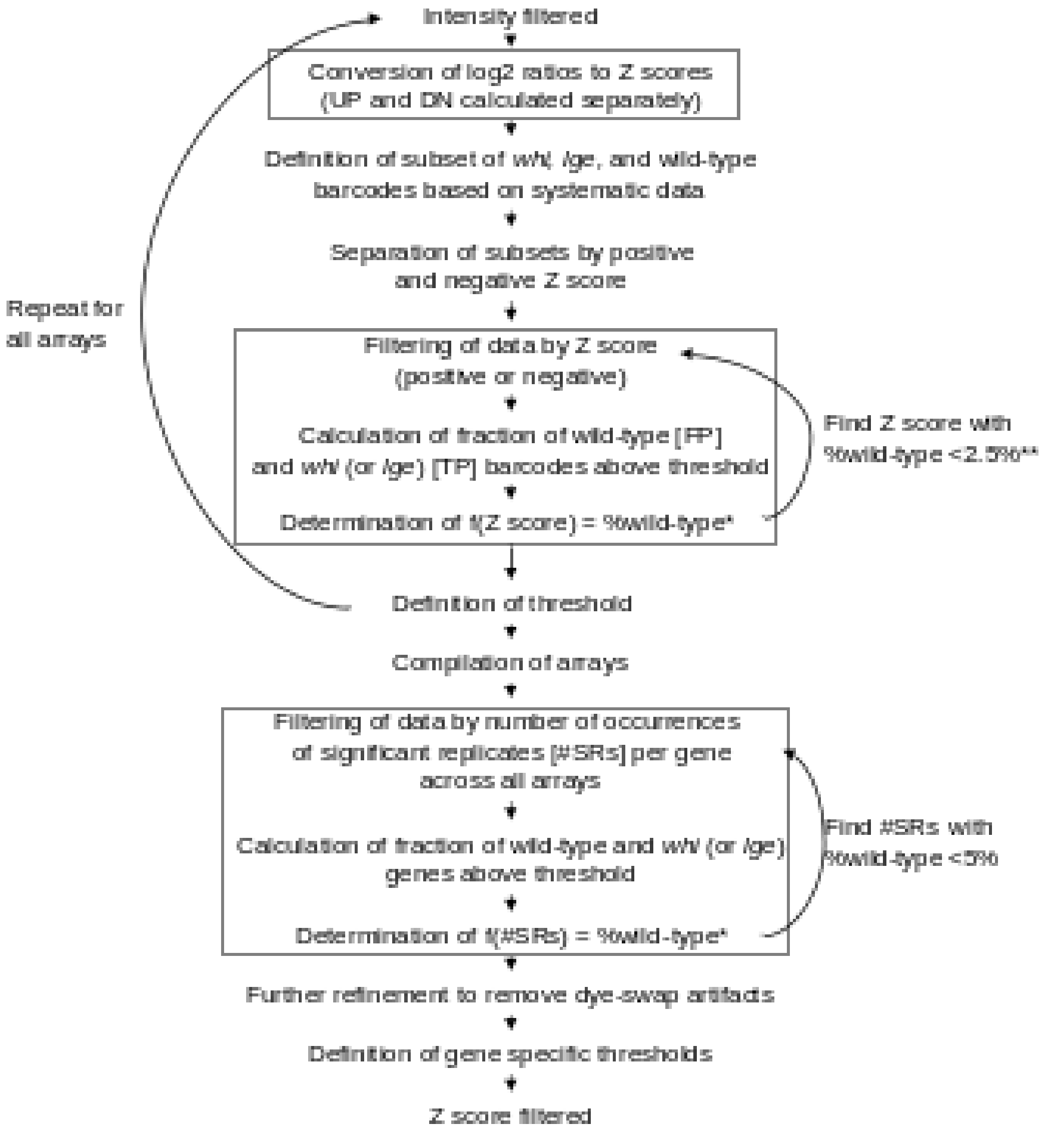

*Rather than maximize the function f(x)=%TP [*wh/* or *lge*]-%FP[wild-type], we chose to weight our approach to minimize false positives, though the thresholds chosen would be similar.

**This corresponds to <5% of total wild-type barcodes, taking into account positive and negative Z scores.
